# Contextualizing the adolescent social brain: Links to social health using data from the Adolescent Brain Cognitive Development Study

**DOI:** 10.1016/j.dcn.2026.101774

**Published:** 2026-06-27

**Authors:** Myles N. Arrington, Johnna R. Swartz, Jeffrey R. Fine, Amanda E. Guyer

**Affiliations:** a Center for Mind and Brain, University of California Davis, 267 Cousteau Place, Davis, CA 95618, USA; b Department of Human Ecology, University of California Davis, 1 Shields Ave, Davis, CA 95616, USA; c Division of Biostatistics, Department of Public Health Sciences, University of California, Davis, CA 95616, USA

**Keywords:** Adolescence, Social health, Social brain, Social information processing, Sex differences

## Abstract

Social health, defined as *adequate quantity and quality of social relationships* (Doyle & Link, 2024), is essential for human well-being. The social brain, a network of regions involved in social cognition, ostensibly facilitates social health; however, few studies assess how social brain function relates to different profiles of social health. We tested this association during early adolescence when close relationships with peers gain increased importance. We used baseline (8- to 11-years old) brain data and year 2 (10- to 13-years old) social health data from 5832 adolescents in the Adolescent Brain Cognitive Development^SM^ (ABCD) Study. We focused on an fMRI task that implicitly elicited emotion processing when viewing faces and applied latent profile analysis on variables associated with peer relationships: the number of friends (close and general), experiences with aggression and victimization, relationships with prosocial and rule-breaking peers, and support from peers. We identified three profiles (“concerning”: 12.96% of sample; “robust”: 33.95%; “selective”: 53.09%). We found a significant interaction between sex and amygdala reactivity to emotional faces, indicating that boys and girls differed in likelihood of belonging to profiles reflecting greater (concerning, robust) vs weaker (selective) peer involvement. Exploratory analyses revealed that associations with other brain regions were only detected when examining individual social health outcomes. This study highlights divergent pathways in how social brain function informs both general social health patterns and individual health outcomes, setting the stage for future research.

## Highlights

• Examined links between social health and eight key social brain regions.
• Latent profile analysis grouped youth as socially selective, robust, or concerning.
• Amygdala activity predicts social health profile membership differentially by sex.
• mOFC, STS, and TPJ are linked to aggression/victimization, not overall profiles.
• Findings suggest future work focus on specific social processes and outcomes.

## Graphical Abstract

**Figure:**
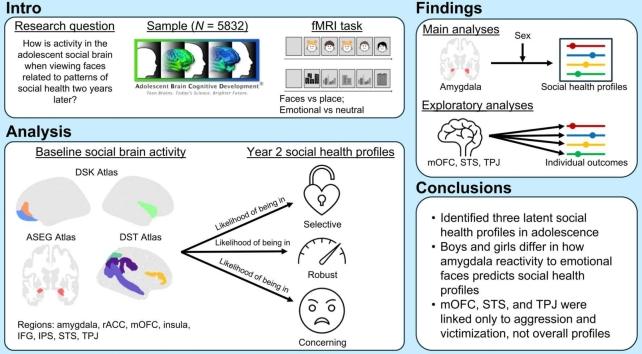

## 1 Introduction

Establishing close social connections is a pressing need for human health (Ford and Highfield, 2016, Woodward et al., 2021). To bolster the likelihood of forming and maintaining important relationships, humans have evolved social-homeostatic systems (Matthews and Tye, 2019) operating at multiple levels, including in the brain. Key regions involved in this process are collectively known as the *social brain* (Brothers, 1990, Dunbar, 1998, Frith, 2007). The social brain undergoes significant developmental changes in adolescence (Blakemore, 2008) that co-occur with changes in social context (Pfeifer and Allen, 2021, Nelson et al., 2016), preparing adolescents for peer relationships (Roisman et al., 2004). However, how the social brain is linked to social connection in adolescence remains understudied. Prior neuroimaging work has focused on multiple independent aspects of social connection, including the number of friends, relationship quality, and experiences with victimization (e.g., Swartz et al., (2019)). Because these studies usually drew on individual measures, understanding of how social brain function relates to relationships across multiple dimensions remains limited.

We leveraged data from the Adolescent Brain and Cognitive Development (ABCD) Study (Volkow et al., 2018) to assess social brain function and social health. The ABCD^Ⓡ^ Study includes both robust neuroimaging data and multiple measures of peer relationships, facilitating a comprehensive assessment of social health. The goal of the present study was to understand how adolescents’ social brain function is associated with how they connect with peers.

### 1.1 The social brain in adolescence

Extensive literature ties the human brain to social behavior. Core regions that support social behavior are collectively referred to as the “social brain” (Brothers, 1990), including regions involved in recognition and social evaluation of others (Blakemore, 2008). Functional activity in these regions has been associated with social relationships (e.g., Kanai et al., (2012); Kwak et al., (2018); Noonan et al., (2018); Von Der Heide et al., (2014)). Engagement of these regions also supports other vital cognitive processes (Atzil et al., 2018, Barrett and Satpute, 2013, Frith, 2007), belying the complexity of cognitive processes involved in social behavior.

Blakemore’s model (2008) focuses on adolescence, which is characterized by significant biobehavioral change (Nelson et al., 2016, Pfeifer and Allen, 2021, Sawyer et al., 2018). These changes impact social brain function, including higher-order cognition and social-emotional processing (Burnett et al., 2009, Burnett et al., 2011, Goddings et al., 2019, Guyer et al., 2009, Scherf et al., 2012, Spielberg et al., 2015, Váša and Romero-Garcia, 2020), and social relationships, including the increasing importance of peers (Nelson et al., 2005, Nelson et al., 2016, Roisman et al., 2004). While there is evidence that these processes are bidirectional (Jarcho et al., 2019, Rudolph et al., 2016, Pfeifer and Allen, 2021, Schriber et al., 2018, Telzer et al., 2018), we focus on how the social brain shapes later social connection (Andrews et al., 2021, Guyer et al., 2023).

Existing findings related to this question encompass disparate processes that are difficult to synthesize without their combined configurations considered in a single study. Some work focuses on qualitative indices (Becht et al., 2021, Güroğlu and Veenstra, 2021), like victimization (Dobbelaar et al., 2025, Du Plessis et al., 2019, Ke et al., 2022, Quinlan et al., 2020, Swartz et al., 2019, Vargas et al., 2019, Visser et al., 2014), time spent with friends (Masten et al., 2012), positive affect (Woods et al., 2020), prosociality (Schreuders et al., 2019), positive experiences (Bates et al., 2025), and trust (Sweijen et al., 2023). Other work focuses on social-relational structure, including social network position (Sijtsma et al., 2023) and number of close friends (Shen et al., 2023). Because few studies examine multiple social connection measures within-persons, a full understanding about their patterns of association with the social brain remains limited. For example, social brain activity might predict not only the number of friendships, but also whether an individual has a large network with high-quality vs low-quality friendships. The goal of this study is to investigate such nuances in the association between the social brain and social relationships in adolescents.

### 1.2 Social health model

The *social health* model offers a framework for more robustly assessing social connection during adolescence. Social health is defined as *adequate quantity and quality of social relationships in a particular context to meet an individual’s need for meaningful human connection* (Doyle and Link, 2024). The focus on relationship properties (quantity, quality) distinguishes the model from approaches that focus on how relationships are perceived (e.g., discrepancy models of loneliness: Cacioppo et al., 2015). Further, the model emphasizes the need to assess social health across multiple measures. Specifically, social health is context-dependent, such that the degree to which relationships are adequate is tailored to the individual’s social needs (Baumeister and Leary, 1995). Therefore, individual differences in one measure may not wholly map on to individual differences in social health. Recent work has highlighted the usefulness of approaches like latent profile analysis that identify patterns across multiple indices to characterize social health (Arrington et al., 2026, Pintos Lobo et al., 2025). This allows for separation of classes that differ across one or more dimensions rather than only a single dimension. Here, we apply a similar approach to assess social health in adolescence.

Our study is guided by prior theory detailing mechanisms by which brain function is linked to social health. For example, the allostatic model argues that brain regions involved in social information processing are also involved in the regulation of internal physiological states (Weissman et al., 2018), which suggests the neural regulation of internal physiology is linked to social health needs (Atzil et al., 2018). The homeostatic model adds that social brain regions are key in detecting social health deficiencies and engaging in behaviors that maintain adequate social health (Matthews and Tye, 2019). We argue that given the social developmental tasks of adolescence, social health needs are increasingly tied to peer relationships (Nelson et al., 2016, Roisman et al., 2004). For example, prior work using data from the ABCD study identified three latent profiles of peer social health: selective, robust, and concerning (Arrington et al., 2026). These patterns were meaningfully associated with other indicators of adjustment, including loneliness and family conflict (Arrington et al., 2026). However, the antecedent neural correlates of these patterns remain unclear, which we aimed to address in this study.

### 1.3 Sex differences

Finally, we were motivated to assess sex differences given extant literature. Prior work using ABCD data indicated that boys were more likely than girls to exhibit concerning social health, while girls were more likely to be selective (Arrington et al., 2026). Other studies indicate that boys display more aggressive behaviors (Card et al., 2008) and provide less support to their peers (van Rijsewijk et al., 2016). A range of mechanisms may explain why sex relates to social health, including differences in attachment style (Ma and Huebner, 2008), social-emotional processing (Oberle et al., 2010), response to peer feedback (Guyer et al., 2014), and susceptibility to peer influence (McCoy et al., 2019). Finally, past research highlights sex-specific trajectories in social brain development (Heitzeg et al., 2018). By including participant sex as an additional predictor, we can investigate sex differences in neurobiological pathways to social health, thereby beginning to reveal how neurobiological characteristics associated with sex are linked to unique social health outcomes. In turn, evidence of sex differences can be used to inform more targeted interventions as a function of sex to improve social health outcomes.

### 1.4 Current study

In the present study, we sought to examine how sex and social brain function predict social health in adolescence. We focused on social brain activity during social information processing using data from the emotional n-back fMRI task (EN-back), collected at baseline (8- to 11-years old). While the EN-back task primarily focuses on working memory, its inclusion of emotional faces allows for implicit assessment of socio-emotional face processing, allowing us to begin to address this research question. Social brain activity when viewing social stimuli is linked with measures like victimization and social support in typically developing adolescents (Hyde et al., 2011, Kellij et al., 2024, Morningstar et al., 2019, Sato et al., 2020, Swartz et al., 2019). Altered social information processing has also been reported in adolescents with major depressive disorder and autism (Fairchild et al., 2014, Hall et al., 2014, Henle-Blom et al., 2015, Nomi and Uddin, 2015, van den Bulk et al., 2014, Weng et al., 2011), which may contribute to impaired social behaviors commonly seen in these populations. Thus, our focus on social processing reflects a key process implicated in the tuning of the social brain for social health. We focused on two contrasts: faces vs places (assessing social vs non-social information processing) and emotional vs neutral faces (assessing valenced social processing).

Given the primacy of social developmental tasks (Roisman et al., 2004), we focused on peer relationships to capture social health using data from assessment year 2 (10- to 13-years old). These measures include the number of friends (close and general), experiences with aggression and victimization, network support, and friendships with prosocial and rule-breaking peers. These measures capture unique dimensions of social health, including both quantity (number of friends, network composition) and quality (experiences with aggression/victimization, network health). We conducted latent profile analysis (LPA) to identify patterns during adolescence and expected to find latent profiles in line with prior work in the ABCD study, which identified “selective,” “robust,” and “concerning” profiles (Arrington et al., 2026).

To define core regions of interest, we followed prior neurodevelopmental theory to focus on regions linked to social-cognitive functions that undergo significant reorganization during adolescence (Blakemore, 2008): the amygdala, anterior cingulate cortex (ACC), inferior frontal gyrus (IFG), insula, intraparietal sulcus (IPS), medial prefrontal cortex (mPFC), posterior superior temporal sulcus (STS), and temporoparietal junction (TPJ). While other areas are involved in social cognition, these eight regions have consistently been implicated in studies of adolescents (Guyer et al., 2008a, Scherf et al., 2013, Silk et al., 2023, Skuse et al., 2003, Sweijen et al., 2023, Vijayakumar et al., 2020, Woods et al., 2020). Our selection of these regions also allowed us to formulate region-specific hypotheses about associations with social health drawing from prior work (Table 1). We predicted that activation in the IFG, IPS, mPFC, STS, and TPJ would predict membership in the robust profile given prior work implicating these regions in emotion processing, processing of social evaluation, mentalizing, and processing of social reward (see Table 1 for references). By contrast, we predicted that activation in the ACC, amygdala, and insula would predict membership in the concerning profile given involvement of these regions in processes related to social exclusion, aggression, and victimization (see Table 1 for references). In cases where the literature diverged,^1^ we drew on research with a design consistent with the current study.

**Table 1:** Study hypotheses.

**A-priori ROI**	**ABCD analog**	**Higher activation predicts…**	**References**
ACC	rACC	Higher likelihood in Concerning	Crowley et al. (2010); Masten et al. (2009)
Amyg.	Amyg.	Higher likelihood in Concerning	Cubillo (2022); Swartz et al. (2019)
IFG	obIFG + opIFG + triIFG	Higher likelihood in Robust	Nakamura et al. (1999)
Insula	Insula	Higher likelihood in Concerning	Ke et al. (2022); Masten et al. (2009)
IPS	IPS and TPS	Higher likelihood in Robust	Narumoto et al. (2001); Rogers et al. (2022)
mPFC	mOFC	Higher likelihood in Robust	Burnett et al. (2009); Somerville et al. (2013)
pSTS	STS	Higher likelihood in Robust	Burnett and Blakemore (2009); Flores et al. (2018)
TPJ	SMG + AG	Higher likelihood in Robust	Burnett and Blakemore (2009); Flores et al. (2018)

Note. Table describes our a-priori selection of adolescent social brain regions based on prior work (Blakemore, 2008), corresponding regions available in ABCD release 6.1, hypotheses regarding associations with social health, and references for those hypotheses. For the ACC, we selected the rostral portion which is usually implicated in social processing. For the IFG, we averaged activation across the three IFG subregions as computed in the pre-processed ABCD data. For the IPS, we used pre-processed ABCD data that collapsed across activation in the intraparietal sulcus and the transverse parietal sulcus. For the mPFC, we selected the ventromedial portion of the PFC to reduce overlap with the dorsomedial portion of the PFC (i.e., rACC). For the TPJ, we averaged activation across the supramarginal gyrus and the angular gyrus as computed in the pre-processed ABCD data. Hypotheses describe main effects of increased activation in each brain region for multinomial regressions where membership in the selective profile is the reference level. (r)ACC = (rostral) anterior cingulate cortex. Amyg. = amygdala. IFG = inferior frontal gyrus. obIFG = orbital part of the IFG. opIFG = opercular part of the IFG. triIFG = triangular part of the IFG. mPFC = medial prefrontal cortex. mOFC = medial orbitofrontal cortex. IPS = intraparietal sulcus. TPS = transverse parietal sulcus. (p)STS = (posterior) superior temporal sulcus. TPJ = temporoparietal junction. SMG = supramarginal gyrus. AG = angular gyrus.

We included sex as a moderator, following work reporting sex differences in social health (Arrington et al., 2026) and in social brain development (Heitzeg et al., 2018). We expected that boys would be more likely to belong to the concerning profile and girls would be more likely to belong to the selective profile, replicating prior work (Arrington et al., 2026) in a smaller and more selective sample. We did not specify *a-priori* hypotheses about interactions between social brain activation and sex.

## 2 Methods

### 2.1 Procedure

The ABCD study is a multi-site longitudinal investigation of adolescent brain development and health outcomes. At baseline, participants included nearly 12,000 adolescents. Recruitment took place at 21 study sites, which were selected to match the national demographics of the United States. Recruitment procedures were designed to slightly oversample ethnic-racial minoritized youth. We used data from release 6.1. For more information about the study design, protocol, and recruitment, please refer to https://abcdstudy.org/scientists/ and Garavan and colleagues (2018). Parents and children provided consent and assent prior to participating in the study.

### 2.2 Participants

The full sample initially included 11,868 adolescents. Participants were excluded if they failed the ABCD fMRI pipeline initial screening (*N* = 4113) or whose assessment was missing (*N* = 72), had significant anatomical variations flagged by a board-certified neuroradiologist (*N* = 291), had < 550 timepoints available contributing to the GLM for EN-back analyses (*N* = 1557), and had beta weight values more than 4 *SD* from the mean (*N* = 3). These criteria were applied to reduce the potential influence of extreme beta weight values on analyses (see Supplemental Materials). The final sample included 5832 adolescents aged 10–13 years old at year 2 (2975 girls; 59.19% non-Hispanic White, 16.79% Hispanic/Latino, 10.84% multiracial, 10.13% Black or African American, 2.02% Asian or Pacific Islander, <1.00% other; see Table 2). Missingness for social health data ranged from 0% to 7% (Table 2); therefore, we applied random-forest imputation via the *tidyLPA* package to maximize sample size in the latent profile analysis. We did not exclude participants based on socioeconomic status, ethnic-racial group, substance use, mental health, or neurodevelopmental history.

**Table 2:** Sample descriptives.

Measure	All Participants		Boys		Girls	
	***N***	**Mean (*****SD*****)**	***N***	**Mean (*****SD*****)**	***N***	**Mean (*****SD*****)**
Close friends	5469	5.99 (*4.94*)	2699	6.03 (*5.26*)	2770	5.95 (*4.61*)
Friends	5469	20.22 (*18.22*)	2699	21.00 (*19.60*)	2770	19.47 (*16.74*)
Aggression	5471	10.22 (*1.78*)	2701	10.42 (*1.91*)	2770	10.04 (*1.63*)
Victimization	5471	12.13 (*3.56*)	2701	12.19 (*3.67*)	2770	12.06 (*3.45*)
Prosocial peers	5444	9.24 (*2.86*)	2683	9.24 (*2.81*)	2761	9.23 (*2.90*)
Rule-breaking peers	5401	3.40 (*1.05*)	2663	3.49 (*1.14*)	2761	3.31 (*0.95*)
Protective support	5832	11.03 (*8.24*)	2857	10.44 (*8.17*)	2975	11.60 (*8.26*)

Note. Table includes sample *N* and mean with standard deviation for each group of participants.

Given concerns about estimating the number of true classes in a population, especially with large sample sizes (Nylund-Gibson and Choi, 2018, Tein et al., 2013), past work indicates a sample of 500 participants is appropriate to detect the true number of classes in a sample (Finch and Bronk, 2011, Spurk et al., 2020). To assess *a-priori* power for multinomial regressions, we conducted a simulation study that indicated a sample of 400 participants was sufficient to detect small-moderate effects in line with our a-priori hypotheses (see Supplemental Materials).

### 2.3 Measures

#### 2.3.1 Peer variables

##### 2.3.1.1 Number of friends (close and overall)

Participants reported social network size by identifying the number of close and non-close (overall) friends in their life (Barch et al., 2018). Close friends were “those you like spending time with, have fun with, and trust.” We summed responses to identify the total number of close and general friends.

##### 2.3.1.2 Experiences with victimization and aggression

Quality of peer interactions was indexed by the Revised Peer Experiences Questionnaire (De Los Reyes and Prinstein, 2004, Prinstein et al., 2001). The measure assesses six features: overt (3 items, e.g. “a teen chased me like he or she was really trying to hurt me”), relational (3 items, e.g. “some teens left me out of an activity or conversation that I really wanted to be included in”), and reputational (3 items, e.g. “A teen tried to damage my social reputation by spreading rumors about me”) victimization as well as overt (3 items, e.g. “I threatened to hurt or beat up another kid”), relational (3 items, e.g. “I did not invite a kid to a party or other social event even though I knew the kid wanted to go”), and reputational (3 items, e.g. “I gossiped about another kid so others would not like him/her”) aggression. Participants indicated how frequently they have had that experience (1 = Never; 2 = Once or twice; 3 = A few times; 4 = About once a week; 5 = A few times a week). We summed across subscales to get total aggression and victimization scores. Cronbach’s alpha indicated that the aggression (ɑ = 0.74) and victimization (ɑ = 0.83) subscales were reliable.

##### 2.3.1.3 Relationships with prosocial and rule-breaking peers

Adolescents reported on who their peers were using the Peer Behavior Profile survey (Bingham et al., 1995). This measure distinguishes involvement with prosocial peers (3 items: how many peers 1) are athletes, 2) go to church, or 3) are good students) and rule-breaking peers (3 items: how many peers 1) skip school, 2) have been suspended from school, or 3) have shoplifted). Participants identify the proportion of their friends that fit into each category (1 = None or almost none; 2 = A few; 3 = Half; 4 = Most; 5 = All or almost all). Items are then summed. Cronbach’s alpha for the prosocial (ɑ = 0.43) and rule-breaking (ɑ = 0.54) subscales indicated moderate reliability. Estimates were slightly higher for girls for both the prosocial (ɑ_boys_ = 0.40, ɑ_girls_ = 0.47) and rule-breaking (ɑ_boys_ = 0.51, ɑ_girls_ = 0.56) subscales. While moderate, these reliability estimates are comparable to prior reports in ABCD (Gonzalez et al., 2021).

##### 2.3.1.4 Peer network health

Adolescents completed the Peer Network Health questionnaire, a modification of the Adolescent Social Network Assessment (ASNA: Mason et al., 2015), to describe peer support. Adolescents answered three items to 1) identify whether their peers have suggested that they stay away from drugs (no = 0, yes = 3), 2) have given any type of support (money, transportation, emotional support: no = 0, yes = 2), or 3) have given any type of encouragement to get or stay involved in prosocial activities (sports, school clubs, religious activities: no = 0, yes = 2). Participants who answered yes on the support or encouragement items also rated the intensity using a 10-point Likert scale (1 = a little, 10 = a lot). We used the composite scale, which sums across all items. To compute reliability, we applied a linear transformation so that for the first item, an answer of no corresponded to 0 and an answer of yes corresponded to 10. For the remaining items, we collapsed across each dichotomous original item and its corresponding Likert item such that an answer of no corresponded to 0 and an answer of yes corresponded to the participant’s answer on the Likert scale. We again found moderate reliability (ɑ = 0.53) for the scale, which was consistent across sex (ɑ_boys_ = 0.53, ɑ_girls_ = 0.53). This estimate converges with prior work using ABCD data (Gonzalez et al., 2021) and may relate to the reduced number of items relative to the full ASNA.

#### 2.3.2 EN-back task

The emotional n-back task, originally designed to assess working memory, consists of 2 runs of 8 blocks each, where 4 blocks consist of 2-back trials and 4 blocks consist of 0-back trials (Barch et al., 2013). Stimuli include emotional faces (positive, negative), neutral faces, and places. On each trial, stimuli are presented for 2 s and followed by a 500-millisecond fixation. Participants were instructed to identify whether the stimulus matched the target. For more information, see Casey et al. (2019).

### 2.4 Data processing and analysis

All processing and analyses were done in R (R Core Team, 2025: version 4.5.1) using RStudio (Posit Team, 2025: version 2025.09.1–401). The full list of packages is included in the Supplemental Materials. Code used in the analysis is available upon request from the authors. We used tabulated data accessed through the NBDC Data Hub (https://www.nbdc-datahub.org/).

For an overview of the fMRI data processing pipeline, see Hagler et al., (2019). We selected regions of interest available in ABCD that overlapped with those comprising the adolescent social brain (Blakemore, 2008: see Table 1). For the ACC, we selected the rostral portion given its role in social-emotional processing (Bush et al., 2000). Because we had no predictions about IFG subregions, we collapsed across the three parts. For the IPS, we used pre-processed data that collapsed across the intraparietal and transverse sulci. Because the rACC, which was already selected as an ROI, can be considered as part of the mPFC (Amodio, Frith, 2006), we selected the medial orbitofrontal cortex (mOFC) as a proxy for the mPFC to ensure coverage across separate social brain regions. This also builds on work highlighting the role of the ventromedial PFC in social cognition (Amodio, Frith, 2006). Lastly, for the TPJ, we collapsed across the supramarginal and angular gyri to ensure coverage across the entire anatomical region corresponding to the intersection of the temporal and parietal lobes (Schurz et al., 2017). For each contrast, we averaged across hemispheres per region.

We winsorized all social health variables at 3 *SD* from the mean to account for extreme values. To account for remaining skew, we used a log transformation for the close and general friend measures, and an inverse transformation for the rule-breaking peers variable. All social health variables were standardized prior to the LPA.

First, we conducted LPA on the social health data and evaluated four different model structures, as available through the *tidyLPA* package, that increase in flexibility of fit and complexity (see Rosenberg et al., 2018). Model 1 assumes that classes differ only in indicator means, model 2 allows classes to differ in indicator means and variances, model 3 restricts indicator variances but freely estimates indicator covariances that are constrained to be equal for each class, and model 6 allows means, variances, and covariances to vary across classes (models 4 and 5 are available only via Mplus: see Rosenberg et al., 2018). We evaluated fits for up to seven profiles. Fit indices included Akaike Information Criterion (AIC) and Bayesian Information Criterion (BIC), which decrease as model fit improves; entropy represents the accuracy in assigning participants to a unique class on a scale of 0–1; class posterior probability is the probability that a participant belongs to the selected class; and class size corresponds to the distribution of profiles across the sample. We set thresholds of identifying a model with comparatively low AIC and BIC, acceptable (> 0.70) or good (> 0.80) values for entropy, acceptable (> 0.80) or good (> 0.90) posterior probabilities across participants and classes, and acceptable (> 5%) or good (> 10%) class sizes for the smallest class, following prior work (Clark and Muthén, 2009, Jung and Wickrama, 2008, Nylund et al., 2007, Pastor et al., 2007). After deciding on the best fit, we used the predicted probabilities to assign each participant to their most likely profile.

Second, we tested how sex and brain function predict social health using multinomial regressions, with profile membership as the dependent variable and social brain function and sex as predictors. We initially aimed to include random intercepts for family ID and scanner manufacturer to account for clustering in the data. Despite attempts using several estimation methods including Bayesian modeling (Bürkner, 2017) and frequentist approaches (Yee, 2010), we encountered persistent issues with model convergence and poor fit. These issues were likely tied to the inclusion of family ID, resulting in many unique cases (i.e., families) in the model. Next, we considered models with only scanner manufacturer as a random effect and found that the models converged. However, we found that estimates for variance due to random effects were low (i.e., < 0.001). Therefore, we report findings from the fixed effects models. The results from the mixed effects models are included in Supplemental Materials.

We ran separate models for each region and contrast, applying an FDR correction to account for multiple comparisons. After fitting each model, we used a likelihood ratio test to estimate the overall significance of the main effects and interactions using nested model comparisons. To evaluate these effects, we set boys in the largest profile as the reference value and computed intercepts and slopes that corresponded to differences in the odds of being in each of the non-reference profiles relative to the odds of being in the largest profile for boys (intercept), and whether this difference differed between boys and girls (slope). We reported odds ratios with confidence intervals as effect sizes. We examined the predicted probabilities of profile membership across the range of activation scores to interpret these effect sizes.

## 3 Results

### 3.1 Descriptives

Mean scores for each social health variable are presented in Table 2. The pattern of correlations among the social health variables (Table 3) aligned with previous work (Arrington et al., 2026). The correlation matrix describing co-activation patterns among the social brain regions is included in the Supplemental Materials.

**Table 3:** Social health correlations.

**Measure**	**1**	**2**	**3**	**4**	**5**	**6**	**7**
1. Close friends	1.00	-	-	-	-	-	-
2. Friends	0.61***	1.00	-	-	-	-	-
3. Aggression	0.04**	0.10***	1.00	-	-	-	-
4. Victimization	0.01	0.04**	0.53***	1.00	-	-	-
5. Prosocial peers	0.21***	0.23***	−0.04**	−0.06***	1.00	-	-
6. Rule-breaking peers	0.12***	0.15***	0.10***	0.09***	0.13***	1.00	-
7. Protective support	0.20***	0.20***	0.05***	0.13***	0.24***	0.11***	1.00

Note. ^*p* < .10; *p < .05; **p < .01; ***p < .001. Cells represent Pearson’s correlation coefficient between social health measures.

### 3.2 Latent profile analysis

The full list of fit indices is included in Table 4. These data suggested a three-profile solution under model 1 was the best fit, aligning with previous work (Arrington et al., 2026). The model 2 and model 6 structures did not converge in our sample, and the solutions for model 3 tended to have unacceptable values for entropy, posterior probabilities, and/or class sizes.

**Table 4:** Latent profile fit indices.

Model	Classes	AIC	BIC	Entropy	Probability [min, max]	Proportion [min, max]
1	1	115874.49	115967.89	1.00	[1.00, 1.00]	[1.00, 1.00]
1	2	113361.89	113508.65	0.62	[0.85, 0.91]	[0.40, 0.60]
**1**	**3**	**110382.65**	**110582.78**	**0.73**	**[0.83, 0.90]**	**[0.10, 0.55]**
1	4	105889.45	106142.96	0.79	[0.81, 1.00]	[0.02, 0.57]
1	5	109423.49	109730.36	0.67	[0.73, 0.90]	[0.10, 0.30]
1	6	104890.37	105250.61	0.70	[0.74, 1.00]	[0.02, 0.30]
1	7	104620.91	105034.52	0.73	[0.60, 1.00]	[0.02, 0.32]
3	1	109840.44	110073.93	1.00	[1.00, 1.00]	[1.00, 1.00]
3	2	108136.54	108423.40	0.91	[0.88, 0.99]	[0.11, 0.89]
3	3	104288.88	104629.11	0.81	[0.89, 1.00]	[0.02, 0.54]
3	4	107535.05	107928.64	0.62	[0.24, 0.94]	[0.07, 0.55]
3	5	101279.09	101726.05	0.81	[0.83, 1.00]	[0.02, 0.49]
3	6	100707.05	101207.38	0.80	[0.80, 1.00]	[0.02, 0.45]
3	7	100724.80	101278.50	0.67	[0.29, 1.00]	[0.02, 0.39]

Note. Cells include fit indices for each possible model identified in the latent profile analysis. Bolded/shaded cells correspond to the selected model fit for this analysis. AIC = Akaike Information Criterion. BIC = Bayesian Information Criterion.

For the model 1 solutions, we found that the third profile provided acceptable fit, was not notably improved in subsequent solutions, and aligned with the results from prior work. Specifically, entropy was acceptable (0.73) with good class sizes (0.10–0.55) and posterior probabilities (0.85–0.91). While the reduction in BIC (Δ_BIC_ = 4439.82) and higher entropy (0.79) signaled some improvement in the four- relative to three-profile solution, this solution resulted in unacceptable class sizes (2.09%). After the four-profile solution, changes in BIC were negligible (e.g., 4–5 Δ_BIC_ = −3587.40) while entropy decreased and posterior probabilities trended below acceptability. Thus, we selected the three-profile solution as the best fit, following prior work (Arrington et al., 2026).

**Fig. 1:**
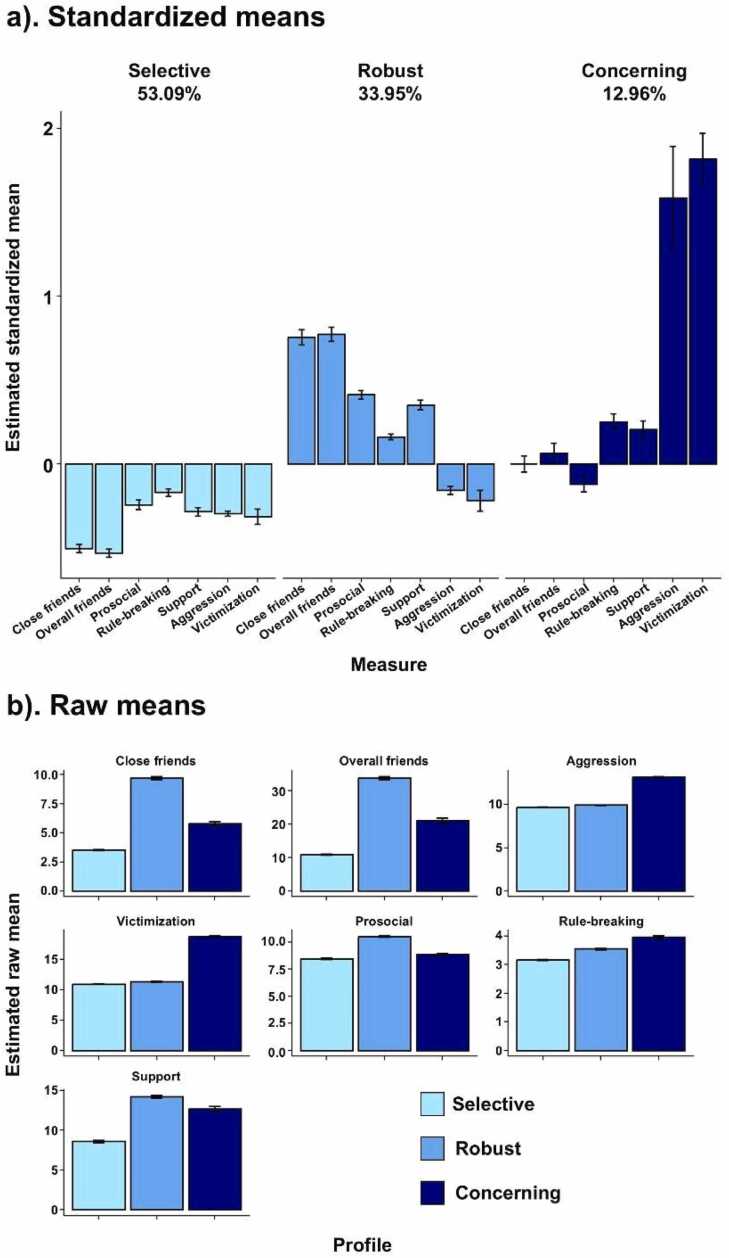
Profile estimated means. Note: Figure presents variable means for the three classes identified in the latent profile analysis solution. Means are visualized using standardized distributions with confidence interval (a) and raw distributions with confidence interval (b) for the variables included in the latent profile analysis. We used these means to characterize and label the three latent profiles. The selective profile (53.09% of the sample) was characterized by mean values that were below the sample mean across variables yet within population norms, especially for the number of friends. The robust profile (33.95% of the sample) was characterized by higher means for numbers of overall and close friends. The concerning profile (12.96% of the sample) was characterized by higher means for aggression, victimization, and rule-breaking peers. All variables are from year 2 of the ABCD Study.

Mean scores for each variable in each profile are visualized in Fig. 1. The first class (“selective”: 53.09% of sample) was characterized by lower scores for each measure, but means were consistent with population estimates, suggesting these adolescents were selective in who they connected with (Arrington et al., 2026). The second class (“robust”: 33.95%) exhibited the highest values for overall friends, close friends, prosocial peers, and peer support, suggesting they had large networks and close relationships. The final class (“concerning”: 12.96%) exhibited the highest values for aggression, victimization, and numbers of rule-breaking peers.

Next, we examined the effect of sex on profile membership. Results indicated that girls were 4 percentage points less likely to be in the concerning profile and 3 percentage points more likely to be in the selective profile (*OR* = 0.71, *p* < 0.001, 95% CI [0.61, 0.84]). This finding converges with prior work (Arrington et al., 2026) and is discussed in more detail in the Supplemental Materials. We also found that girls had significantly lower amygdala (*b* = −0.02, *p* = 0.009, 95% CI [−0.03, −0.00], Cohen’s *d* = 0.06) and IPS (*b* = −0.02, *p* = 0.017, 95% CI [−0.03, −0.00], Cohen’s *d* = 0.06) activity to faces vs places compared to boys, albeit with a small effect size. No other sex differences in brain activation emerged across regions and contrasts (see Supplemental Materials).

### 3.3 Does social brain function predict social health?

Results of the likelihood ratio test (LRT) and the corresponding effects from each multinomial regression are reported in Table 5. Although the main effect of emotional vs neutral amygdala activity was non-significant (*Χ*^2^(2) = 3.92, *p*_*raw*_ = 0.141, *p*_*adjust*_ = 0.281), we found a significant interaction with sex (*Χ*^2^(2) = 9.16, *p*_*raw*_ = 0.010, *p*_*adjust*_ = 0.031). This interaction indicated sex differences in the likelihood of belonging to the concerning relative to the selective profile (*OR* = 2.34, *p*_raw_ = 0.011, *p*_adjust_ = 0.029, 95% CI [1.21, 4.50]), and the robust relative to the selective profile (*OR* = 1.71, *p*_raw_ = 0.023, *p*_adjust_ = 0.058, 95% CI [1.08, 2.73]). These effects indicate the relation between amygdala activation and social health differs significantly by sex: the magnitude of sex differences are estimated as 2.34 times larger for the concerning versus selective contrast and 1.71 times larger for the robust versus selective contrast.

**Table 5:** Results from multinomial regressions.

**Effect**		**Sel vs Rob**	**Sel vs Conc**
	***Χ***^**2**^	***OR*****[LL, UL]**	***OR*****[LL, UL]**
**rACC**			
*E vs N*			
Brain	1.77	0.96 [0.72, 1.28]	0.77 [0.53, 1.13]
Int.	4.60	1.22 [0.81, 1.84]	1.86 [1.04, 3.31]^2^
*F vs P*			
Brain	0.37	0.99 [0.74, 1.32]	1.11 [0.76, 1.62]
Int.	0.29	0.90 [0.60, 1.34]	0.94 [0.53, 1.67]
**Amyg.**			
*E vs N*			
Brain	3.92	0.78 [0.56, 1.09]	0.68 [0.44, 1.05]
**Int.**	**9.16**	**1.71 [1.08, 2.73]**^**2**^	**2.34 [1.21, 4.50]**
*F vs P*			
Brain	0.66	1.06 [0.77, 1.46]	1.19 [0.78, 1.83]
Int.	2.75	0.71 [0.45, 1.11]	0.70 [0.37, 1.33]
**IFG**			
*E vs N*			
Brain	6.08^1^	0.75 [0.55, 1.04]	0.62 [0.41, 0.95]^2^
Int.	5.08	1.55 [0.99, 2.44]	1.72 [0.91, 3.25]
*F vs P*			
Brain	0.03	0.99 [0.72, 1.36]	1.03 [0.68, 1.57]
Int.	0.08	0.95 [0.61, 1.46]	1.02 [0.55, 1.88]
**Insula**			
*E vs N*			
Brain	2.12	0.93 [0.63, 1.37]	0.68 [0.41, 1.14]
Int.	0.67	1.19 [0.69, 2.04]	1.31 [0.60, 2.85]
*F vs P*			
Brain	0.14	1.08 [0.73, 1.58]	1.05 [0.63, 1.76]
Int.	0.22	0.89 [0.52, 1.52]	0.89 [0.42, 1.91]
**IPS**			
*E vs N*			
Brain	0.29	0.98 [0.69, 1.40]	1.12 [0.71, 1.78]
Int.	1.17	0.95 [0.58, 1.53]	0.69 [0.35, 1.36]
*F vs P*			
Brain	0.07	0.97 [0.70, 1.34]	0.95 [0.62, 1.45]
Int.	0.12	1.07 [0.69, 1.66]	1.08 [0.58, 2.01]
**mOFC**			
*E vs N*			
Brain	4.09	0.96 [0.82, 1.13]	0.81 [0.65, 0.99]^2^
Int.	5.88	1.18 [0.95, 1.48]	1.44 [1.05, 1.97]^2^
*F vs P*			
Brain	2.12	0.98 [0.84, 1.15]	1.15 [0.93, 1.41]
Int.	0.50	1.07 [0.86, 1.33]	1.08 [0.80, 1.48]
**STS**			
*E vs N*			
Brain	5.30	0.77 [0.52, 1.14]	0.56 [0.34, 0.94]^2^
Int.	1.02	1.24 [0.72, 2.14]	1.39 [0.64, 3.02]
*F vs P*			
Brain	1.42	1.15 [0.80, 1.66]	0.86 [0.53, 1.38]
Int.	2.11	0.98 [0.59, 1.63]	1.65 [0.81, 3.37]
**TPJ**			
*E vs N*			
Brain	1.58	0.83 [0.55, 1.25]	0.73 [0.43, 1.26]
Int.	0.74	1.24 [0.70, 2.20]	0.92 [0.41, 2.07]
*F vs P*			
Brain	0.30	1.04 [0.70, 1.54]	0.89 [0.53, 1.50]
Int.	0.50	1.07 [0.62, 1.86]	1.32 [0.61, 2.88]

Note. Cells correspond to model output for each model in the analysis. E vs N = emotional vs. neutral contrast in the emotional n-back task. F vs P = face vs. place contrast in the emotional n-back task. rACC = rostral anterior cingulate cortex. Amyg. = amygdala. IFG = inferior frontal gyrus. IPS = intraparietal sulcus. mOFC = medial orbitofrontal cortex. STS = superior temporal sulcus. TPJ = temporoparietal junction. Sel = selective profile. Rob = robust profile. Conc = concerning profile. Brain = main effect of brain activity in the multinomial regression; region and contrast as indicated. Int. = interaction between participant sex and the indicated measure of brain activity in the multinomial rShaded and bolded cells correspond to significant effects. ^1^ = Results from likelihood ration tests that did not survive correction. ^2^ = Results from probing coefficients that did not survive correction.

**Fig. 2:**
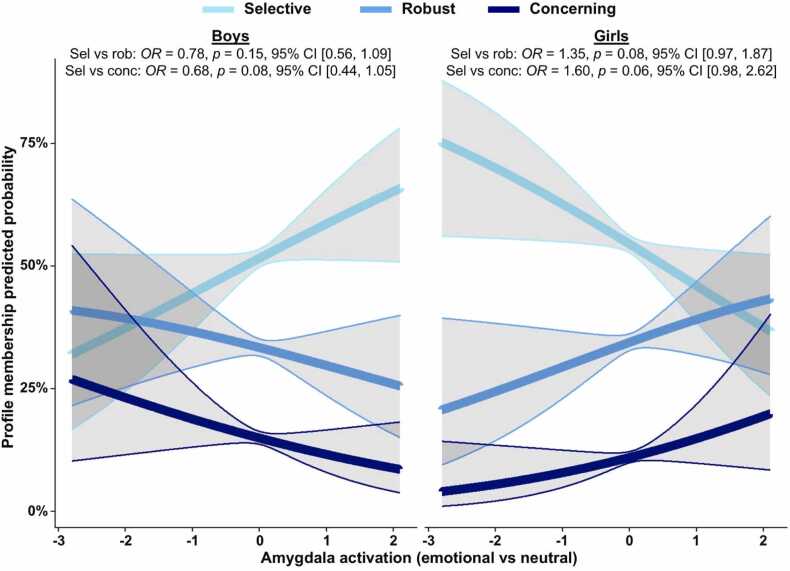
Longitudinal associations between amygdala activation and later social health by sex. Note: Figure shows results from the multinomial regression testing how baseline amygdala activation in response to emotional vs neutral faces and participant sex interact to predict the probability of membership in each of the latent social health profiles at year 2. In the multinomial regressions, each index of social brain activity was entered as a predictor of membership in each of the three latent social health profiles alongside sex. Membership in the selective profile was set as the reference. Each line shown in the figure shows the predicted probability (i.e., likelihood on a total range of 0–100%) of a participant belonging to each of the three latent social health profiles across the range of social brain activity. Simple effects corresponding to the selective vs robust and selective vs concerning contrasts are reported for each sex group.

To interpret this, we ran post-hoc analyses using separate models for each sex group and found that for boys, higher amygdala activation was associated with reduced odds of belonging to the robust profile (*OR* = 0.78, *p* = 0.148, 95% CI [0.56, 1.09]) and the concerning profile (*OR* = 0.68, *p* = 0.084, 95% CI [0.44, 1.05]), although neither effect was statistically significant. For girls, higher amygdala activation was associated with increased odds for the robust profile (*OR* = 1.35, *p* = 0.076, 95% CI [0.97, 1.87]) and the concerning profile (*OR* = 1.60, *p* = 0.062, 95% CI [0.98, 2.62]), although neither effect was statistically significant. Therefore, even though we were unable to detect effects that met criteria for significance, the post-hoc analyses indicated that the significant interaction between participant sex and amygdala activation can be attributed to differences in the direction of effects for each group, with boys tending (non-significantly) to be less likely to belong to the robust and concerning profiles (versus selective), while the inverse effect was true for girls (Fig. 2).

We also found evidence implicating emotional vs neutral IFG activation; however, this effect did not survive correction (see Supplemental Materials). No other significant main or interaction effects emerged from our analyses involving other social brain regions, in contrast to our hypotheses.

### 3.4 Exploratory analysis for individual social health outcomes

Given 1) results only implicated one social brain region, and 2) prior reports of associations with social brain activity for individual social health outcomes, we conducted exploratory analyses using individual outcomes as the dependent variable. We hypothesized that evidence of associations may suggest that links between social brain activity and social health may operate via outcome-specific pathways. We tested this using linear regressions following an identical modeling approach as the multinomial regressions (see Supplemental Materials for methods).

The full results from this analysis are included in the Supplemental Materials. Three main effects of brain activation on social health survived FDR correction across all tests: main effects for emotional vs neutral STS (*b* = −0.32, *p*_*raw*_ = 0.007, *p*_*adjust*_ = 0.046, 95% CI [−0.54, −0.09]) and TPJ (*b* = −0.38, *p*_*raw*_ = 0.002, *p*_*adjust*_ = 0.014, 95% CI [−0.62, −0.14]) activity in predicting aggression, and a main effect for faces vs places mOFC activity in predicting victimization (*b* = 0.28, *p*_*raw*_ = 0.003, *p*_*adjust*_ = 0.018, 95% CI [0.10, 0.46]). Taken together, these results reveal specific associations for social brain regions that were not implicated in the profile-level analyses.

## 4 Discussion

In the present study, we examined how social brain function may predict social health during early adolescence. This builds on a conceptual framework describing the process of neurobiological and behavioral social reorientation towards peers during adolescence, through which adolescents experience changes in brain function in preparation for more complex and intimate relationships with their peers (Nelson et al., 2005, Nelson et al., 2016). As a result, a core hypothesis of this framework is that emerging social brain function facilitates peer relationships in support of adolescent social health. The goal of this work was to directly test this hypothesis by examining how social brain activity when viewing faces at baseline predicts social health profile membership two years later in a sample of early adolescents.

### 4.1 Social health in adolescence

To assess social health in this study, we applied a latent profile analysis on seven variables indexing various aspects of peer relationships: the number of close friends, the number of overall friends, experiences with aggression and victimization, network support and health, and friendships with prosocial and rule-breaking peers. This analysis follows previous work with a larger sub-sample of ABCD participants (*N* = 10,050: Arrington et al., 2026), which examined the same seven measures and identified a solution with three profiles as the best fit for the data. While the sample in the present study was approximately half the size due to rigorous fMRI screening, results from the LPA converged with this prior work. Specifically, 1) the three-profile solution was the best fit for the data, 2) the indicator means for the three profiles approximate the means of the larger sample, and 3) the class sizes only slightly diverge from the larger sample. However, across these two studies, social health has only been examined using LPA at the two-year follow-up. Thus, it is unknown whether these three profiles change with age in following years, which is an important consideration for future social health research.

### 4.2 Social brain as a predictor of social health

By assessing how activity in social brain regions in early adolescence predicts social health profile membership two years later, we leveraged the methodological uniqueness of the ABCD study to hypothesize longitudinal links between brain function and social health. In addition, we examined two separate aspects of social-emotional processing: the contrast between social (i.e., faces) and non-social (i.e., places) information, and the contrast between valenced (emotional) and non-valenced (neutral) social information.

Only one brain region was linked to social health profile membership. Specifically, we found sex differences in how amygdala activation to emotional vs neutral faces predicted social health profile membership. Post-hoc analyses supported the conclusion that this interaction was likely related to differences in the direction of effects for each group, although we were unable to detect these effects at a significant level. For boys, higher activation tended to predict reduced likelihood of belonging to the robust or concerning profiles (versus selective profile), but for girls, higher activation tended to predict increased likelihood of belonging to these profiles. Our limited ability to detect significant effects for these trends likely reflects discrepancies in sample size between profiles in the multinomial regressions, which are magnified when stratified by sex (e.g., approximately 400 boys in the concerning profile compared to 1500 boys in the selective profile), as prior work indicates that such differences may limit power (Peduzzi et al., 1996). Thus, it is possible a larger overall sample would have provided more power to detect these effects in a subset of the sample (i.e., each sex group).

Broadly, this finding aligns with neurodevelopmental work that highlights the relevance of the amygdala as a social-emotional salience detector (Adolphs, 2010, Amaral, 2003, Hariri et al., 2002, Santos et al., 2011, Vrtička et al., 2013) that undergoes critical structural and functional development during adolescence (Goddings et al., 2014, Scherf et al., 2013). The centering of our findings on the emotional vs neutral contrast aligns with literature emphasizing developmental sensitivity to emotionally-salient information during adolescence (Casey et al., 2019, Nelson et al., 2016) and may highlight the centrality of emotional processing in peer relations in this age range (Crone and Dahl, 2012). Our finding of sex differences suggests that the role of functional development in the amygdala as a predictor of social outcomes varies by sex. Interestingly, the direction of effects were consistent within each sex group across both “positive” (robust) and “negative” (concerning) profiles of social health, where both profiles can be characterized as including higher peer involvement than the selective profile. As a result, amygdala reactivity to emotional faces may index the degree of engagement with peer relationships more broadly, rather than predicting whether peer involvement manifests as positive (robust) or negative (concerning) outcomes. Thus, the interaction suggests that the role of the amygdala in supporting social engagement across multiple indices during early adolescence differs by sex, which may relate to prior work finding sex-specific lateralization in adolescent amygdala activity during face processing (Schneider et al., 2011) and sex-differential amygdala activation in response to threat in adults (McClure et al., 2004). Our post-hoc analyses indicated that for girls, heightened amygdala reactivity tended to predict greater peer involvement, including opportunities for both robust and concerning social health, while for boys, heightened activity tended to predict less peer involvement (although not statistically significant); more work with larger samples may be needed to assess whether these effects can be detected at a significant level.

Several mechanisms may be involved in the link between social brain activity and social health during adolescence, with pubertal development representing one key candidate mechanism. For example, prior work has linked sex differences in amygdala activation (Heitzeg et al., 2018) to sex-specific pubertal development in adolescence, impacting not only social processing (Bürger et al., 2023, Rose and Rudolph, 2006, Stroud et al., 2002) but also factors related to peer relationships like internalizing symptoms (Naninck et al., 2011, Rohde et al., 2013) and reward sensitivity (Alarcón et al., 2017, Barendse et al., 2024). This is particularly relevant given reported differences in pubertal timing and tempo, particularly in earlier stages of adolescence (Marceau et al., 2011). At the same time, recent work in the ABCD sample found limited evidence for associations between pubertal development and emotion processing that diverge by sex (Qian et al., 2026), suggesting that future work exploring puberty as a potential explanatory mechanism take care to draw from a rigorous theoretical foundation to ensure that puberty is measured at the appropriate level (e.g., pubertal stage vs hormonal activity vs pubertal timing) and in relation to the appropriate task. Our results may also relate to sex differences in socialization, especially as it pertains to learning to process social information. For example, boys may be socialized to withhold intimacy and distance themselves from their peers (Way, 2011), which may shape how youth learn to process social information, including the neural correlates of face processing (Proverbio, 2017). Given that both neurobiological (i.e., puberty) and socialization factors are likely involved in the development of social health, our finding of divergent pathways related to amygdala activation prompt the need for further investigation into the biological and social mechanisms that may explain this finding.

To more comprehensively assess links between social brain activity and social health, we also explored associations with individual social health outcomes, with the goal of determining whether other brain regions were implicated in specific social health outcomes rather than profile-level social health patterns. After applying FDR correction across all tests, these analyses revealed that emotional vs neutral STS and TPJ activation were negatively linked to aggression, and that faces vs places mOFC activation was positively linked to victimization. Prior work indicates that youth with conduct disorder exhibit hypoactivation in the TPJ relative to controls when viewing the intentional infliction of pain (Decety et al., 2009); these findings suggest that aggressive behavior is linked to atypical neural responses to emotional stimuli in regions supporting mentalizing. By contrast, OFC activation has been linked to the ability to make social predictions (Rudebeck and Murray, 2014) and engage in self-monitoring (Beer et al., 2006); thus, the link between mOFC activation during general face processing and victimization experiences may reflect heightened neural sensitivity to social evaluation, potentially indicating vulnerability to peer victimization.

Taken together, the limited number of robust associations across profile-level and individual-level analyses suggests that links between social brain activation during the EN-back task and real-world peer relationships two years later may be more modest than initially hypothesized. This may reflect several factors. For example, our findings may be related to the nature of the fMRI paradigm used in this study. The functional contrasts assessed here reflected social information processing, which is closely tied to amygdala development during adolescence (Scherf et al., 2013). By contrast, regions like the mOFC, STS, and TPJ are more closely linked to other social behavior processes like mentalizing (Blakemore, 2008, Rudebeck and Murray, 2014, Schurz et al., 2014). Therefore, subsequent work should also consider tasks that elicit additional social processes, including mirroring and mentalizing (Alcalá-López et al., 2019, Frith and Frith, 2006, Van Overwalle and Baetens, 2009), regulation of social-emotional distress (Masten et al., 2009, Masten et al., 2013, Petrovic and Ingvar, 2002), and processing of social novelty (Guyer et al., 2009, Woods et al., 2020). This may help capture unique influences of multiple regions underlying separate social processes, how one region may be involved in separate processes supporting social health (Crone and Fuligni, 2020), and how a network of regions supporting a social behavior (Schurz et al., 2014) may be linked to social health profile membership. Other potential reasons for our findings may include the two-year temporal lag in time, the explicit focus of the task on working memory rather than social-emotional processing, or the complexity of social health development that may be lost when coalescing across multiple indicators. These considerations inform our recommendations for future research.

### 4.3 Limitations and future directions

First, given that the measures used in this study were not specifically designed to assess social health, it is possible we may have found more robust associations if using different measures to capture social health. Still, our analysis of social health replicates the results of a prior study, which found evidence for ecological validity of this approach by observing associations with family conflict and loneliness in the ABCD sample (Arrington et al., 2026). It will be important for future research to operationalize alternate ways to assess social health (i.e., ecological momentary assessment: Baek et al., 2025). Also, some of the social health indices had only moderate reliability. We chose to retain these summary scores given prior reports that scores from these scales behave as expected in association with other dimensions of youth adjustment (Gonzalez et al., 2021), to reduce the number of indicators included in the LPA resulting in a more parsimonious model, and to maintain conceptual links between related items. Even so, future research should consider either alternate approaches to verify the validity of these measures (e.g., McDonald’s omega) or the use of item-level indices.

Further, the primary focus of the emotional n-back task is on working memory, such that activity elicited during the task may reflect memory-related more than social processes. At the same time, inclusion of emotional faces implicitly requires emotion processing so that participants accurately track identity across expressions (Casey et al., 2018, Morningstar and Burns, 2025); therefore, the task offers a valuable way to begin to assess social information processing. Given trends from our data specifically implicating emotional processing, future work should consider using tasks with an explicit focus on emotions.

Our findings may also be influenced by data processing procedures. For example, we only had access to data that collapsed across the intraparietal and the transverse parietal sulci, which may have limited our ability to detect an effect specifically for the IPS. Similarly, we collapsed across posterior and anterior portions of the STS and defined the TPJ anatomically rather than functionally. Therefore, associations with social brain regions may have emerged with differently-defined social brain ROI. In addition, other social brain regions may also be implicated in this association, like the fusiform face area (Alarcón et al., 2020, Burnett et al., 2011). Similarly, other approaches could be used to assess the social brain, including evaluation of size or structure (Bickart et al., 2011) or examination of functional connectivity (McCormick et al., 2018). Thus, the present study leaves room for future researchers to further examine how the social brain may be implicated in social health development.

Given our focus on peers, we also suggest examination of stimuli that differ in developmental salience. For example, prior work highlights heightened neural sensitivity to peer-relevant information during adolescence (Guyer et al., 2008b, Güroğlu et al., 2008, Lau et al., 2012, Saxbe et al., 2015), which is particularly relevant given our focus on peer relationships. Therefore, it is possible that an fMRI task that elicited responses to faces that varied by age (peer vs adult-aged) may have revealed more robust associations with social health. More work will be needed to help evaluate these alternative hypotheses.

We did not assess other features of adolescence that might further clarify these findings given the research questions that drove this study. Prior work has highlighted how pubertal development is related to changes in the social brain (Goddings et al., 2012, Scherf et al., 2012), which may ultimately impact peer relations (Blakemore, 2008, Pfeifer and Allen, 2021). Individual differences in the unfolding of pubertal development over time may be key to understanding how the social brain shapes social health, including sex-specific pathways. For example, while early pubertal timing is related to accelerated brain development for girls and boys, there appear to be sex differences in regions implicated in this acceleration (Dehestani et al., 2023). Given that pubertal timing is also related to friendship selection (Franken et al., 2016) and peer victimization (Skoog and Kapetanovic, 2022), further investigation into puberty may help clarify how the social brain is tied to social health during this period.

We did not assess change over time for social brain function or social health, limiting longitudinal inferences from these findings. This is important because prior work indicates there may be a high degree of variability in these factors over time (social brain: van den Bulk et al., 2013; social health: Ferguson et al., 2022; Poulin and Chan, 2010). We suggest that future researchers also consider links between changes in social brain function and social health during adolescence to understand the developmental processes that may underlie this association.

Lastly, our strict screening protocol may limit the generalizability of these findings, such that the findings largely reflect adolescents who are more likely to provide high-quality fMRI data (see Supplemental Materials).

### 4.4 Conclusions

The goal of this study was to examine potential links between social brain activation and social health profiles in early adolescence. Guided by prior work, we focused on activity in eight a-priori regions of the social brain while viewing emotional and neutral faces, and applied latent profile analysis on several measures related to adolescent peer relationships. Results from the LPA converged with prior work, identifying selective, robust, and concerning patterns of peer social health. Further, we found a significant interaction between sex and amygdala activation in predicting social health profiles, suggesting that boys and girls tended to differ in likelihood of belonging to profiles reflecting greater vs less peer involvement. In addition to offering validation of the latent profile classifications in an ABCD sample with restrictive screening compared to prior work, the current study also offers important considerations for future work examining links between social brain activity and social health. Specifically, these results suggest that future researchers consider mechanisms by which sex may be related to the social brain-health link, such as pubertal development or sex-related socialization, use tasks that explicitly elicit specific social processes, including emotion processing, and formulate hypotheses about how these processes support both general social health patterns and specific health outcomes. Findings from the present study contribute new understanding to the social reorientation processes and set the stage for future work on linking social brain function to long-term social health across the lifespan.

## CRediT authorship contribution statement

**Arrington Myles N:** Writing – review & editing, Writing – original draft, Visualization, Software, Methodology, Formal analysis, Conceptualization. **Swartz Johnna R:** Writing – review & editing. **Jeffrey R. Fine:** Writing – review & editing, Visualization, Software. **Guyer Amanda E:** Writing – review & editing, Writing – original draft, Supervision, Project administration, Funding acquisition, Conceptualization.

## Declaration of Competing Interest

The authors declare that they have no known competing financial interests or personal relationships that could have appeared to influence the work reported in this paper.

## Data Availability

Code used in the analysis is available upon request from the authors. To access ABCD data, visit https://www.nbdc-datahub.org/ to apply for access to the NBDC Data Hub.

## Footnotes

1 For example, some studies link increased prefrontal activity to increased sensitivity or proclivity to social situations (Burnett et al., 2009, Somerville et al., 2013), suggesting a positive association with membership in the selective or robust profiles. Other studies indicate that dampened activity reflects dampened inhibitory control and therefore closer relationships with peers (Morningstar et al., 2019), suggesting a negative association with membership in the selective or robust profiles.
