# Supplementary material for: Contextualizing the adolescent social brain: Links to social health using data from the Adolescent Brain Cognitive Development Study
Source: Dev Cogn Neurosci. 2026 Jun 27;80:101774. doi: 10.1016/j.dcn.2026.101774 (PMC13332478; doi:10.1016/j.dcn.2026.101774)
Supplement: Supplementary file 1 — Supplementary material [file mmc1.docx]

**S1. Participant screening**

The full sample initially included 11,868 adolescents. An overview of the screening procedure is provided in Figure S1. Participants were excluded if they failed the ABCD fMRI pipeline initial screening (*N* = 4,113) or whose assessment was missing (*N* = 72), indicating they had failed at least one initial screening criterion including quality control at multiple stages (e.g., Freesurfer, manual post-processing), adequate registration to structural images, and identification of derived results. Given that we still observed extreme beta weight values (scores > ±5 *SD* from the mean), we applied additional criteria including exclusion of participants who were flagged by a board-certified neuroradiologist for incidental findings during scanning (e.g., anatomical variations: *N* = 291), had <550 timepoints available contributing to the GLM for n-back analyses (*N* = 1,557), and who still exhibited extreme beta weight values after applying these criteria (*N* = 3). After applying these criteria, the data more approximately resembled a normal distribution (see Figure S2). These criteria have also been applied in other ABCD analyses (O’Brien et al., 2020; Rosenberg et al., 2020). The final sample size of 5832 adolescents is comparable to recent studies that have also used ABCD EN-back data (Barendse et al., 2026; Gaillard et al., 2025; Kuhney et al., 2026; Morningstar & Burns, 2025). Histograms for the social health data are included in Figure S3.

As an additional supplemental analysis, we also examined potential factors that may have been related to the likelihood of an individual participant passing these screening criteria. Using logistic regressions in the full ABCD sample, we tested whether participant demographics (sex, ethnic-racial group, age), social health (using the seven peer relationship variables, and social brain activation (across the eight ROI for each selected contrast) predicted likelihood of inclusion (1) or exclusion (0). For models using continuous data as predictors, we winsorized the data to limit the effect of extreme scores.

Results indicated that girls (*OR =* 1.29, *p* < 0.001, 95% CI [1.20, 1.39]), White non-Hispanic youth (*OR*_range_ = 0.39 – 0.79, all *p* < 0.030, all 95% CI [0.35, 0.97]), and older adolescents (*OR =* 1.45, *p* < 0.001, 95% CI [1.37, 1.54]) were more likely to be included than their counterparts. We also found that aggression (*OR =* 0.97, *p* = 0.002, 95% CI [0.95, 0.99]), victimization (*OR =* 0.98, *p* < 0.001, 95% CI [0.97, 0.99]), and proportion of rule-breaking peers (*OR =* 0.89, *p* < 0.001, 95% CI [0.87, 0.92]) predicted decreased likelihood of inclusion, while proportion of prosocial peers (*OR =* 1.08, *p* < 0.001, 95% CI [1.06, 1.09]) and network health (*OR =* 1.01, *p* = 0.001, 95% CI [1.00, 1.01]) predicted increased likelihood of inclusion. Lastly, study inclusion was positively predicted by amygdala faces vs places activation (*OR =* 1.21, *p* < 0.001, 95% CI [1.08, 1.35]) and negatively predicted by IPS faces vs places activation (*OR =* 0.76, *p* < 0.001, 95% CI [0.67, 0.86]).

Taken together, these findings are largely consistent with the fact that our primary screening criteria revolved around fMRI data quality control. For example, prior work suggests that younger and male participants tend to move more in the scanner, which may alter data quality (Engelhardt et al., 2017; Hodgson et al., 2017). Likewise, prior work in the ABCD sample suggests that minoritized youth are more likely to exhibit excessive motion (Ramduny et al., 2026). Likewise, psychopathology (e.g., anxiety) also reduces the likelihood of obtaining high-quality imaging data (Eijlers et al., 2023; Hercules et al., 2025) and is also linked to worse social health (Chung et al., 2019; Erath et al., 2007). The associations between brain activation patterns and inclusion probability should be interpreted cautiously, as they likely reflect data quality considerations rather than true population-level differences in neural function. Participants with extreme or atypical activation patterns (in either direction) were more likely to be excluded through our quality control procedures, which prioritized retaining data with reliable signal and minimal artifacts. Notably, only two activation predictors emerged from 16 possible predictors, suggesting that there was not a consistent pattern across all measures of brain activity.

While the exclusion criteria we used help ensure reliable inferences about brain-behavior associations, they do limit the generalizability of our findings. Specifically, the sample included in this study best reflects adolescents who can successfully complete fMRI protocols with minimal motion and adequate data quality. Even so, this work helps researchers begin to understand links that tie social brain activity to social health. Future research should prioritize methodological innovations to reduce contextual barriers to participation (Falk et al., 2013), including the use of prospective motion correction, shorter scan protocols, and acclimation procedures.

**S2. Simulation study**

To determine the power to detect effects in line with our a-priori hypotheses, we set up a simulation study to design a dataset consistent with our predictions. Our hypotheses (see Table 1 in manuscript) were that increased social brain activation during the n-back task would be associated with increased likelihood of belonging to the concerning profile or the robust profile, depending on the region. Broadly, we expected small-moderate effect sizes for these relationships; we conducted the simulation study based on this prediction, specifically focusing on our predictions regarding the concerning profile. Because we ran multiple models in testing our hypotheses, we then used a false discovery rate correction to account for multiple comparisons. The code used in this simulation is available upon request and is adapted from the University of Virginia’s StatLab resources (Ford, 2023).

First, we specified a uniform distribution of social brain activation scores with values ranging from -3 to 3 and a distribution of categorical values (e.g., “boy”, “girl”). Next, we defined starting values for the effect that these variables and their interaction have on the log odds coefficients corresponding to the two non-reference levels of a three-level multinomial variable. We specified intercepts so that the group proportions would approximate the distribution of profiles in the observed data (i.e., first group approximates the selective profile, second group approximates the robust profile, third group approximates the concerning profile) and starting values for the effect of sex such that there would be minimal sex differences for membership in the second profile and significant sex differences for membership in the third profile. We also specified coefficients such that there would be a negligible main effect of brain activation for the log odds of the second level and there would be a significant main effect of brain activation for the log odds of the third level, corresponding to our hypothesis that increased brain activation would predict increased likelihood of belonging to the concerning profile. Lastly, while we did not have a-priori hypotheses regarding a possible interaction with sex, we used starting values that would reveal no interaction in the case of the second level and a small negative interaction in the case of the third level. Using these starting values, we then generated a simulated dataset with 2500 observations by calculating predicted probabilities of class membership based on the values in the brain and sex distributions.

To verify that this simulated dataset was consistent with our hypotheses, we then ran a multinomial regression and interpreted the effects. The associations among these variables are visualized in Figure S4. Results indicated that there was no effect of sex (*OR* = 1.02, *p* = 0.851, 95% CI [0.85, 1.21]), no effect of brain activation (*OR* = 1.00, *p* = 0.980, 95% CI [0.93, 1.08]), and no interaction (*OR* = 1.01, *p* = 0.907, 95% CI [0.91, 1.11]) when contrasting membership in the first group compared to membership in the second group. However, we found effects for sex (*OR* = 0.59, *p* < 0.001, 95% CI [0.44, 0.78]), brain activation (*OR* = 1.41, *p* < 0.001, 95% CI [1.27, 1.57]), and the interaction (*OR* = 0.87, *p* = 0.094, 95% CI [0.74, 1.02]) for the first group compared to the third group. The effects of sex were in line with prior work evaluating profile membership (see Arrington et al., 2026), and the main effect of brain activation for the third level was consistent with our hypotheses regarding the concerning profile. Therefore, we concluded that these were appropriate starting values with which to estimate power.

Next, we evaluated the power to detect these effects over a range of sample sizes. To do so, we set up a routine that would replicate 500 simulated datasets for each designated sample size. For each simulation, we conducted a likelihood ratio test to determine whether a model that included sex, brain activation, and the interaction performed significantly better than a model that included only sex as a predictor (i.e., excluding brain activation and the interaction). Then, across the 500 simulations for each designated sample size, we calculated the proportion of ANOVAs that revealed that the model with the interaction was significantly better than the model with only sex. This allowed us to determine the likelihood of detecting these effects at a significance level of alpha = 0.05 (i.e., power) across a range of sample sizes. We evaluated sample sizes ranging from 25 to 500 in intervals of 25. Results indicated that by a sample size of 400, power exceeded 80% as estimated from the simulated power curve (Figure S5). In other words, our analyses required a minimum of 400 participants to detect our predicted effects.

Ford, C. (2023). Simulating Multinomial Logistic Regression Data. UVA Library StatLab. https://library.virginia.edu/data/articles/simulating-multinomial-logistic-regression-data.

**S3. R packages**

We used the following packages in R: *car* (Fox & Weisberg, 2019: version 3.1-3), *corrplot* (Wei & Simko, 2024: version 0.95), *cowplot* (Wilke, 2024: version 1.1.3), *effects* (Fox & Hong, 2010; Fox & Weisberg, 2019: version 4.2-2), *interactions* (Long, 2024: version 1.2.0), *lme4* (Bates et al., 2015: version 1.1-37), *lmerTest* (Kuznetsova et al., 2017: version 3.1-3), *mclogit* (Elff, 2022: version 0.9.6), *nnet* (Venables & Ripley, 2002: version 7.3-19), *psych* (Revelle, 2024: version 2.4.12), *rstatix* (Kassambara, 2023: version 0.7.2), *tidyLPA* (Rosenberg et al., 2018: version 1.1.0), *tidymodels* (Kuhn & Wickham, 2020: version 1.2.0), and *tidyverse* (Wickham et al., 2019: version 2.0.0).

**S4. Coactivation patterns among social brain regions**

We tested co-activation patterns across the eight regions for each contrast via correlation matrices, with the goal of determining whether participants’ task-evoked activations were coordinated or independent across the social brain. This allowed us to determine the degree to which between-person differences in one social brain region covaried with between-person differences for the other seven regions. To do so, we submitted beta weights indexing activation for each of the eight social brain ROI to a Pearson’s product correlation matrix, separately for the emotional vs neutral faces and the faces vs places contrasts. These matrices are displayed in Figure S6.

We observed strong consistency in functional activation for both contrasts among the insula, inferior frontal gyrus, intraparietal sulcus, superior temporal sulcus, and temporoparietal junction (*r* > 0.60), indicating a high level of shared variance in activation among these regions. By contrast, the amygdala, rACC, and mOFC were moderately correlated with each other and the other brain regions (0.25 < *r* < 0.65), suggesting a higher level of functional independence for these three regions.

In summary, the eight social brain regions differed in how closely the functional response to the task aligned with the functional response of other regions. It is important to note that this analysis is not equivalent to a functional connectivity analysis, which is derived from within-persons analysis of how activation in separate regions is linked across time. Rather, this analysis used a between-persons approach (based on average activation patterns across a task) to understand how average activation in one region is correlated with average activation in another region. Even so, this finding sets the stage for future research to examine how social brain connectivity may be associated with social health (Gabard-Durham et al., 2014).

**S5. Effects of sex on profile membership and social brain activation**

Prior to our primary models that examined the effects of sex and brain activation on profile membership, we also examined the main effect of sex on profile membership via a multinomial regression and the effects of sex on patterns of brain activation for each contrast and region using linear regressions. Results from the multinomial regression indicated that sex was not associated with the odds of belonging to the robust profile relative to the selective profile (*OR* = 0.99, *p* = 0.895, 95% CI [0.89, 1.11]). However, sex was a significant predictor of the odds of belonging the concerning profile relative to the selective profile, such that girls had a 30% reduction in the odds of belonging to the concerning relative to the selective profile compared to boys: *OR* = 0.71, *p* < 0.001, 95% CI [0.61, 0.84]. The predicted probabilities indicated that girls were 4 percentage points less likely to belong to the concerning profile (Figure S7). This finding replicated the results of a prior analysis with a larger ABCD subsample (Arrington et al., 2026). These estimates were consistent across the analyses that included brain activation and an interaction between sex and activation (i.e., the analyses reported in the main text; see Table S1). Regarding the effects of sex on social brain activation, we found a significant effect sex differences in amygdala (*b* = -0.02, *p* = 0.009, 95% CI [-0.03, -0.00], Cohen’s *d* = 0.07) and IPS (*b* = -0.02, *p* = 0.017, 95% CI [-0.03, -0.00], Cohen’s *d* = 0.06) faces vs places activation, indicating that girls were slightly less reactive to faces compared to boys. No other marginal or significant effects emerged (see Table S2).

We also examined effects of sex on individual social health outcomes. These effects align with those reported in prior work (Arrington et al., 2026), with girls reporting fewer aggressive experiences and more protective support, and are reported in Table S3.

**S6. Uncorrected multinomial regression effects**

In our primary analyses examining whether participant sex and social brain activity interact to predict social health patterns, we found one additional effect that was significant prior to FDR correction. Specifically, we found a main effect of emotional vs neutral IFG activation (*Χ*^2^(2) = 6.08, *p_raw_* = 0.048, *p_adjust_* = 0.127) indicating that increased activation was related to a decrease in likelihood of belonging to the concerning relative to the selective profile (*OR* = 0.62, *p*_raw_ = 0.028, *p*_adjust_ = 0.069, 95% CI [0.41, 0.95]). The predicted probabilities corresponding to these effects are visualized in Figure S8.

**S7. Multinomial regression output with concerning profile as reference**

Given mixed evidence for associations with social health profiles in our primary analyses, we also explored whether social brain activity was related to social health profile membership when we set the concerning profile as the reference rather than the selective profile. Using this approach allows us to compute concerning vs selective (as reported in the manuscript) and concerning vs robust contrasts (in contrast to the manuscript), thereby evaluating whether social brain activity is related to the likelihood of *not being in the concerning profile*. Results from the multinomial regressions are presented in Table S4. Because results from the likelihood ratio tests and the effects for the concerning vs selective profiles are comparable to what we reported in the manuscript, the table only reports results from the concerning vs robust contrast. Across the models, we did not find significant evidence for social brain activity predicting differences in membership for these profiles. Therefore, we did not find evidence for social brain activation reliably predicting the difference in odds of belonging to the concerning relative to the robust profile.

**S8. Testing the association between social brain activity and individual social health outcomes**

Given 1) results implicated only one social brain region, and 2) prior reports of associations with social brain activity for individual social health outcomes, we conducted exploratory analyses using individual outcomes as the dependent variable. We tested this using linear regressions following an identical modeling approach as the multinomial regressions. Specifically, we specified linear models where each social brain activity indicator and participant sex interacted to predict social health outcomes. We ran separate models for each social health outcome and social brain activity score.

Although we identified several main effects and interactions linking social brain activity to individual outcomes (see Table S5), only three survived FDR correction: main effects for emotional vs neutral STS (*b* = -0.32, 95% CI [-0.54, -0.09], *p_raw_* = 0.007, *p_adjust_* = 0.046) and TPJ (*b* = -0.38, 95% CI [-0.62, -0.14], *p_raw_* = 0.002, *p_adjust_* = 0.014) activity in predicting aggression, and a main effect for faces vs places mOFC activity in predicting victimization (*b* = 0.28, 95% CI [0.10, 0.46], *p_raw_* = 0.003, *p_adjust_* = 0.018). Taken together, these findings suggest that aggression and victimization were primarily related to reactivity to emotional faces in regions involved in thinking about others, including making predictions about behavior, social perception, and mentalizing (Blakemore, 2008; Rudebeck & Murray, 2014; Schurz et al., 2014). This pattern aligns with prior work suggesting that stronger neural responses to emotional social stimuli may support emotion regulation capacities that reduce aggressive behavior (Morawetz et al., 2016). Interestingly, however, prior research also specifically implicates amygdala activation in relation to aggression/victimization (e.g. Swartz et al., 2019); we were unable to detect associations with the amygdala in relation to aggression/victimization here.

In contrast, the influence of insular and IFG activation on aggression, positive associations between STS and TPJ activation and prosocial peer affiliation, and the divergent associations with amygdala and IFG faces vs places activation across close friends, prosocial peers, and network health, did not survive correction. We suggest that these findings should be interpreted with caution pending replication in samples with less restrictive screening and in analyses with more specific hypotheses about links between brain function and social behavior. The corrected findings highlight that emotional processing in mentalizing regions (STS, TPJ) shows the most robust associations with peer relationships during early adolescence.

Main effects related to sex aligned with those presented in Table S3.

**S9. Mixed-effects multinomial regression output**

As described in the main text, we initially set out to run mixed effects multinomial regressions with fixed effects of sex, social brain activation, and the interaction alongside random effects of family (to account for siblings) and scanner ID (to account for possible differences in protocol). Despite attempts using several estimation methods including Bayesian modeling via the *brms* package (Bürkner, 2017) and frequentist approaches via the *VGAM* package (Yee, 2010), we encountered persistent issues with model convergence and poor fit. These issues were seemingly tied to the inclusion of family ID, which likely arose from the large number of unique cases (e.g., families) that needed to be estimated in the model. Next, we considered models with only scanner manufacturer as a random effect and found that the models converged. However, we found that estimates for variance due to random effects were low (i.e., < 0.001) for each of the models. For this reason, we report the results from fixed effects multinomial regressions in the main text. We have also included here the estimates derived from the mixed effects models, as described in Table S6. We fit the models using the *mblogit*() function from the *mclogit* package and specified a random intercept that varied by scanner ID using a quasi-likelihood approach to approximate the random effects structure (i.e., method = “PQL”) with standard maximum likelihood estimation for the fixed effects (i.e., estimator = “ML”). Note that across these models, all reported effects were consistent with the analyses reported in the main text (compare with Table 5 in main text). This suggests that the inclusion of random effects did not substantially change the results.

**Supplemental References**

Barendse, M. E. A., Fine, J. R., Taylor, S. L., Swartz, J. R., Shirtcliff, E. A., Yoon, L., Farnsworth, I., Tully, L. M., & Guyer, A. E. (2026). Frontal-limbic mediated implicit cognitive control of emotion in the transition to adolescence. *Cognitive, affective & behavioral neuroscience*, *26*(1), 89–103. https://doi.org/10.3758/s13415-025-01363-4.

Bates, D., Mächler, M., Bolker, B., Walker, S. (2015). Fitting Linear Mixed-Effects Models Using lme4. *Journal of Statistical Software*, 67(1), 1–48.

Chung, J. E., Song, G., Kim, K., Yee, J., Kim, J. H., Lee, K. E., & Gwak, H. S. (2019). Association between anxiety and aggression in adolescents: a cross-sectional study. *BMC pediatrics*, *19*(1), 115.

Eijlers, R., Blok, E., White, T., Utens, E. M., Tiemeier, H., Staals, L. M., ... & Dierckx, B. (2023). Internalizing and externalizing behaviors in school-aged children are related to state anxiety during magnetic resonance imaging. *Aperture Neuro*, 3, 1-10. doi: 10.52294/​001c.85071.

Elff, M. (2022) mclogit: Multinomial logit models, with or without random effects or overdispersion. R package version 0.9.6. https://CRAN.R-project.org/package=mclogit.

Engelhardt, L. E., Roe, M. A., Juranek, J., DeMaster, D., Harden, K. P., Tucker-Drob, E. M., & Church, J. A. (2017). Children’s head motion during fMRI tasks is heritable and stable over time. *Developmental cognitive neuroscience*, *25*, 58-68.

Erath, S. A., Flanagan, K. S., & Bierman, K. L. (2007). Social anxiety and peer relations in early adolescence: Behavioral and cognitive factors. *Journal of abnormal child psychology*, *35*(3), 405-416.

Fox, J., & Hong, J. (2010). Effect Displays in R for Multinomial and Proportional-Odds Logit Models: Extensions to the effects Package. *Journal of Statistical Software*, *32*, 1–24. https://doi.org/10.18637/jss.v032.i01.

Fox, J., & Weisberg, S. (2019). *An R companion to applied regression, Third edition*. Sage: Thousand Oaks, CA. https://www.john-fox.ca/Companion/.

Gabard-Durnam, L. J., Flannery, J., Goff, B., Gee, D. G., Humphreys, K. L., Telzer, E., Hare, T., & Tottenham, N. (2014). The development of human amygdala functional connectivity at rest from 4 to 23 years: a cross-sectional study. *NeuroImage*, *95*, 193–207. https://doi.org/10.1016/j.neuroimage.2014.03.038.

Gaillard, M., Jones, S. A., Kliamovich, D., Flores, A. L., & Nagel, B. J. (2025). Negative life events during early adolescence are associated with neural deactivation to emotional stimuli. *Brain and Cognition*, 187, 106303.

Hercules, K., Liu, Z., Christofilea, E., Wei, J., Venegas, G., Ciocca, O., ... & Ibrahim, K. (2025). Transdiagnostic Symptom Domains Have Distinct Patterns of Association With Head Motion During Multimodal Imaging in Children. *Biological Psychiatry Global Open Science*, *5*(4), 100506.

Hodgson, K., Poldrack, R. A., Curran, J. E., Knowles, E. E., Mathias, S., Göring, H. H., ... & Glahn, D. C. (2017). Shared genetic factors influence head motion during MRI and body mass index. Cerebral Cortex, 27(12), 5539-5546.

Kassambara, A. (2023). rstatix: Pipe-Friendly Framework for Basic Statistical Tests. R package version 0.7.2. https://CRAN.R-project.org/package=rstatix.

Kuhn, M., & Wickham, H. (2020). *Tidymodels: A collection of packages for modeling and machine learning using tidyverse principles*. https://www.tidymodels.org.

Kuhney, F. S., Mittal, V. A., & Damme, K. S. (2026). Anterior Cingulate Cortex Reactivity to Social Stimuli Marks Individual Differences to Peer Victimization on Internalizing Symptoms in Adolescence. *JAACAP Open*. doi: https://doi.org/10.1016/j.jaacop.2025.12.006.

Kuznetsova, A., Brockhoff, P. B., Christensen, R. H. B. (2017). lmerTest Package: Tests in Linear Mixed Effects Models. *Journal of Statistical Software*, *82*(13), 1-26. doi: 10.18637/jss.v082.i13.

Long, J.A. (2024). interactions: Comprehensive, User-Friendly Toolkit for Probing Interactions. R package version 1.2.0, URL: https://cran.r-project.org/package=interactions.

Morawetz, C., Kellermann, T., Kogler, L., Radke, S., Blechert, J., & Derntl, B. (2016). Intrinsic functional connectivity underlying successful emotion regulation of angry faces. *Social cognitive and affective neuroscience*, *11*(12), 1980–1991. https://doi.org/10.1093/scan/nsw107.

Morningstar, M., & Burns, J. A. (2025). Probing Puberty as a Source of Developmental Change in Neural Response to Emotional Faces in Early Adolescence. *Developmental Psychobiology*, 67(2), e70037.

O’Brien, K. J., Barch, D. M., Kandala, S., & Karcher, N. R. (2020). Examining specificity of neural correlates of childhood psychotic-like experiences during an emotional n-back task. *Biological Psychiatry: Cognitive Neuroscience and Neuroimaging*, 5(6), 580-590.

Ramduny, J., Uddin, L. Q., Vanderwal, T., Feczko, E., Fair, D. A., Kelly, C., & Baskin-Sommers, A. (2026). Representing Brain-Behavior Associations by Retaining High-Motion Minoritized Youth. *Biological psychiatry. Cognitive neuroscience and neuroimaging*, *11*(2), 155–170. https://doi.org/10.1016/j.bpsc.2025.01.014.

Revelle, W. (2024). psych: Procedures for Personality and Psychological Research, Northwestern University, Evanston, Illinois, USA, https://CRAN.R-project.org/package=psych.

Rose, A. J., & Rudolph, K. D. (2006). A review of sex differences in peer relationship processes: Potential trade-offs for the emotional and behavioral development of girls and boys. *Psychological Bulletin, 132*(1), 98–131. https://doi.org/10.1037/0033-2909.132.1.98.

Rosenberg, J. M., Beymer, P. N., Anderson, D. J., Van Lissa, C. J., & Schmidt, J. A. (2018). tidyLPA: An R Package to Easily Carry Out Latent Profile Analysis (LPA) Using Open-Source or Commercial Software. *Journal of Open Source Software*, 3(30), 978, https://doi.org/10.21105/joss.00978.

Rosenberg, M. D., Martinez, S. A., Rapuano, K. M., Conley, M. I., Cohen, A. O., Cornejo, M. D., ... & Casey, B. J. (2020). Behavioral and neural signatures of working memory in childhood. *Journal of Neuroscience*, 40(26), 5090-5104.

Venables, W. N. & Ripley, B. D. (2002). Modern Applied Statistics with S. Fourth Edition. Springer, New York. ISBN 0-387-95457-0.

Wei, T., & Simko, V. (2024). R package 'corrplot': Visualization of a Correlation Matrix (Version 0.95). Available from https://github.com/taiyun/corrplot.

Wickham, H., Averick, M., Bryan, J., Chang, W., McGowan, L. D. A., François, R., ... & Yutani, H. (2019). Welcome to the Tidyverse. *Journal of open source software*, *4*(43), 1686. doi: https://doi.org/10.21105/joss.01686.

Wilke, C. (2024). cowplot: Streamlined Plot Theme and Plot Annotations for 'ggplot2'. R package version 1.1.3, https://CRAN.R-project.org/package=cowplot.

**Table S1. Effects of sex on profile membership**

| **Effect** | **Sel vs Rob** | **Sel vs Conc** |
| --- | --- | --- |
|  | *OR* [LL, UL] | *OR* [LL, UL] |
| rACC |  |  |
| *E vs N* | 0.99 [0.89, 1.11] | 0.72 [0.61, 0.84] |
| *F vs P* | 0.99 [0.89, 1.11] | 0.71 [0.61, 0.84] |
| Amyg. |  |  |
| *E vs N* | 0.98 [0.87, 1.09] | 0.70 [0.59, 0.82] |
| *F vs P* | 1.06 [0.92, 1.23] | 0.77 [0.62, 0.94] |
| IFG |  |  |
| *E vs N* | 0.99 [0.89, 1.04] | 0.72 [0.61, 0.84] |
| *F vs P* | 0.99 [0.89, 1.13] | 0.71 [0.61, 0.84] |
| Insula |  |  |
| *E vs N* | 0.99 [0.89, 1.11] | 0.71 [0.61, 0.84] |
| *F vs P* | 1.00 [0.89, 1.14] | 0.72 [0.61, 0.84] |
| IPS |  |  |
| *E vs N* | 0.99 [0.89, 1.11] | 0.71 [0.61, 0.83] |
| *F vs P* | 1.00 [0.87, 1.15] | 0.72 [0.59, 0.88] |
| mOFC |  |  |
| *E vs N* | 0.99 [0.89, 1.10] | 0.71 [0.61, 0.84] |
| *F vs P* | 0.99 [0.88, 1.11] | 0.71 [0.60, 0.84] |
| STS |  |  |
| *E vs N* | 0.99 [0.88, 1.11] | 0.71 [0.61, 0.84] |
| *F vs P* | 0.99 [0.88, 1.11] | 0.70 [0.59, 0.82] |
| TPJ |  |  |
| *E vs N* | 0.99 [0.89, 1.11] | 0.71 [0.61, 0.84] |
| *F vs P* | 0.99 [0.89, 1.11] | 0.72 [0.61, 0.84] |

Note: Cells contain *OR* with 95% confidence interval from multinomial regressions testing sex x brain activation effects on profile membership. The effects of brain activation and the interaction are reported in Table 4 in the main text. All selective vs concerning contrasts were significant at the level of *p* < 0.05. E vs N = emotional vs. neutral contrast in the emotional n-back task. F vs P = face vs. place contrast in the emotional n-back task. rACC = rostral anterior cingulate cortex. Amyg. = amygdala. IFG = inferior frontal gyrus. IPS = intraparietal sulcus. mOFC = medial orbitofrontal cortex. STS = superior temporal sulcus. TPJ = temporoparietal junction. Sel = selective profile. Rob = robust profile. Conc = concerning profile.

**Table S2. Effects of sex on brain activation**

|  | **Emotional vs neutral** | | | **Face vs place** | | | |  |
| --- | --- | --- | --- | --- | --- | --- | --- | --- |
| **Region** | *b [LL, UL]* | *p* | *d* | | *b [LL, UL]* | *p* | *d* | |
| rACC | -0.00 [-0.02, 0.01] | 0.622 | 0.01 | | -0.00 [-0.02, 0.01] | 0.639 | 0.01 | |
| Amyg. | -0.01 [-0.02, 0.01] | 0.281 | 0.03 | | **-0.02 [-0.03, -0.00]** | **0.009** | **0.07** | |
| IFG | 0.00 [-0.01, 0.02] | 0.591 | -0.01 | | -0.01 [-0.02, 0.01] | 0.365 | 0.02 | |
| Ins. | -0.00 [-0.01, 0.01] | 0.621 | 0.01 | | -0.01 [-0.02, 0.00] | 0.234 | 0.03 | |
| IPS | 0.00 [-0.01, 0.01] | 0.696 | -0.01 | | **-0.02 [-0.02, -0.00]** | **0.017** | **0.06** | |
| mOFC | -0.01 [-0.03, 0.02] | 0.583 | 0.01 | | -0.01 [-0.04, 0.01] | 0.347 | 0.02 | |
| STS | 0.00 [-0.01, 0.01] | 0.640 | -0.01 | | 0.01 [-0.01, 0.02] | 0.232 | -0.03 | |
| TPJ | 0.00 [-0.01, 0.01] | 0.400 | -0.02 | | 0.01 [-0.01, 0.02] | 0.331 | -0.03 | |

Note: Cells contain coefficient *beta* with 95% confidence interval, *p*-value, and Cohen’s *d* from linear regressions testing sex differences in brain activation. Shaded and bolded cells correspond to significant effects. rACC = rostral anterior cingulate cortex. Amyg. = amygdala. IFG = inferior frontal gyrus. IPS = intraparietal sulcus. mOFC = medial orbitofrontal cortex. STS = superior temporal sulcus. TPJ = temporoparietal junction.

**Table S3. Effects of sex on social health outcomes**

| **Outcome** | ***b [LL, UL]*** | ***p*** | ***d*** |
| --- | --- | --- | --- |
| Close friends | 0.02 [-0.01, 0.05] | 0.239 | -0.03 |
| Friends | -0.02 [-0.06, 0.02] | 0.272 | 0.03 |
| **Aggression** | **-0.38 [-0.47, -0.28]** | **<0.001** | **0.21** |
| Victimization | -0.13 [-0.32, 0.06] | 0.167 | 0.03 |
| Prosocial peers | -0.01 [-0.17, 0.14] | 0.851 | 0.01 |
| Rule-breaking peers | -0.01 [-0.01, 0.00] | 0.104 | 0.04 |
| **Protective support** | **1.16 [0.74, 1.58]** | **<0.001** | **-0.14** |

Note: Cells contain coefficient *beta* with 95% confidence interval, *p*-value, and Cohen’s *d* from linear regressions testing sex differences in social health outcomes. Shaded and bolded cells correspond to significant effects.

**Table S4. Results from multinomial regressions with concerning profile as reference**

| **Effect** | **Concerning vs Robust**  ***OR* [LL, UL]** |
| --- | --- |
| **rACC** |  |
| *E vs N* |  |
| Brain | 1.24 [0.83, 1.85] |
| Int. | 0.66 [0.36, 1.21] |
| *F vs P* |  |
| Brain | 0.89 [0.60, 1.33] |
| Int. | 0.95 [0.52, 1.73] |
| **Amyg.** |  |
| *E vs N* |  |
| Brain | 1.15 [0.72, 1.81] |
| Int. | 0.73 [0.37, 1.46] |
| *F vs P* |  |
| Brain | 0.89 [0.57, 1.40] |
| Int. | 1.01 [0.51, 1.97] |
| **IFG** |  |
| *E vs N* |  |
| Brain | 1.21 [0.77, 1.89] |
| Int. | 0.91 [0.46, 1.77] |
| *F vs P* |  |
| Brain | 0.96 [0.62, 1.50] |
| Int. | 0.93 [0.49, 1.77] |
| **Insula** |  |
| *E vs N* |  |
| Brain | 1.37 [0.79, 2.37] |
| Int. | 0.90 [0.40, 2.05] |
| *F vs P* |  |
| Brain | 1.02 [0.59, 1.76] |
| Int. | 1.00 [0.45, 2.23] |
| **IPS** |  |
| *E vs N* |  |
| Brain | 0.88 [0.54, 1.43] |
| Int. | 1.37 [0.67, 2.84] |
| *F vs P* |  |
| Brain | 1.02 [0.65, 1.61] |
| Int. | 0.99 [0.52, 1.91] |
| **mOFC** |  |
| *E vs N* |  |
| Brain | 1.19 [0.95, 1.49] |
| Int. | 0.82 [0.59, 1.15] |
| *F vs P* |  |
| Brain | 0.85 [0.65, 1.07] |
| Int. | 0.99 [0.71, 1.37] |
| **STS** |  |
| *E vs N* |  |
| Brain | 1.37 [0.80, 2.37] |
| Int. | 0.89 [0.39, 2.01] |
| *F vs P* |  |
| Brain | 1.35 [0.82, 2.24] |
| Int. | 0.59 [0.28, 1.26] |
| **TPJ** |  |
| *E vs N* |  |
| Brain | 1.14 [0.64, 2.01] |
| Int. | 1.34 [0.57, 3.17] |
| *F vs P* |  |
| Brain | 1.17 [0.67, 2.03] |
| Int. | 0.81 [0.36, 1.84] |

Note. Cells correspond to estimates corresponding to odds ratios of belonging to the concerning vs the robust profiles for each model in the analysis. E vs N = emotional vs. neutral contrast in the emotional n-back task. F vs P = face vs. place contrast in the emotional n-back task. rACC = rostral anterior cingulate cortex. Amyg. = amygdala. IFG = inferior frontal gyrus. IPS = intraparietal sulcus. mOFC = medial orbitofrontal cortex. STS = superior temporal sulcus. TPJ = temporoparietal junction.

**Table S5. Effects of brain activation on individual outcomes**

| **Outcome** | **Region** | **Contrast** | **Effect** | ***b [LL, UL]*** | ***p_raw_*** | ***p_adjust_*** |
| --- | --- | --- | --- | --- | --- | --- |
| Close friends | Amyg. | Emo v neut | Interaction | 0.14 [0.01, 0.27] | 0.041 | 0.225 |
| Close friends | IFG | Emo v neut | Interaction | 0.13 [0.00, 0.26] | 0.046 | 0.247 |
| Close friends | Amyg. | Face v place | Interaction | -0.14 [-0.27, -0.01] | 0.033 | 0.187 |
| Aggression | Insula | Emo v neut | Main effect | -0.27 [-0.50, -0.05] | 0.014 | 0.087 |
| Aggression | IFG | Emo v neut | Main effect | -0.24 [ -0.43, -0.05] | 0.014 | 0.086 |
| **Aggression** | **STS** | **Emo v neut** | **Main effect** | **-0.32 [-0.54, -0.09]** | **0.007** | **0.046** |
| **Aggression** | **TPJ** | **Emo v neut** | **Main effect** | **-0.38 [-0.62, -0.14]** | **0.002** | **0.014** |
| Victimization | STS | Emo v neut | Main effect | -0.52 [-0.98, -0.07] | 0.025 | 0.152 |
| Victimization | TPJ | Emo v neut | Main effect | -0.52 [-1.00, -0.05] | 0.033 | 0.185 |
| **Victimization** | **mOFC** | **Face v place** | **Main effect** | **0.28 [0.10, 0.46]** | **0.003** | **0.018** |
| Prosocial | Amyg. | Face v place | Interaction | -0.77 [-1.37, -0.16] | 0.013 | 0.084 |
| Prosocial | IFG | Face v place | Interaction | -0.66 [-1.24, -0.08] | 0.026 | 0.157 |
| Prosocial | STS | Face v place | Main effect | 0.34 [0.01, 0.69] | 0.043 | 0.234 |
| Prosocial | TPJ | Face v place | Main effect | 0.40 [0.03, 0.77] | 0.032 | 0.185 |
| Prot. support | Amyg. | Emo v neut | Interaction | 1.95 [0.23, 3.67] | 0.027 | 0.157 |

Note: Cells contain coefficient *beta* with 95% confidence interval, raw *p*-value, and corrected *p*-value from linear regressions testing how sex and brain activation interact to predict individual social health outcomes. FDR correction was applied across all tests computed in this exploratory analysis. Main effects correspond to main effects of brain activation, and interactions correspond to interactions between brain activation and participant sex. Shaded and bolded cells correspond to significant effects after correction. Prot. support = protective support. Amyg. = amygdala. IFG = inferior frontal gyrus. mOFC = medial orbitofrontal cortex. STS = superior temporal sulcus. TPJ = temporoparietal junction. Emo v neut = emotional vs neutral faces contrast in the EN-back task. Face v place = faces vs places contrast in the EN-back task.

**Table S6. Results from mixed effects multinomial regressions**

| **Effect** | **Sel vs Rob** | **Sel vs Conc** |
| --- | --- | --- |
|  | ***OR* [LL, UL]** | ***OR* [LL, UL]** |
| rACC |  |  |
| *E vs N* |  |  |
| Brain | 0.96 [0.72, 1.28] | 0.77 [0.53, 1.13] |
| Int. | 1.22 [0.81, 1.84] | 1.86 [1.04, 3.31] |
| *F vs P* |  |  |
| Brain | 0.99 [0.74, 1.32] | 1.11 [0.76, 1.62] |
| Int. | 0.90 [0.60, 1.34] | 0.94 [0.53, 1.67] |
| Amyg. |  |  |
| *E vs N* |  |  |
| Brain | 0.78 [0.56, 1.09] | 0.68 [0.44, 1.05] |
| Int. | 1.71 [1.08, 2.73] | 2.34 [1.21, 4.50] |
| *F vs P* |  |  |
| Brain | 1.06 [0.77, 1.46] | 1.19 [0.78, 1.83] |
| Int. | 0.71 [0.45, 1.11] | 0.70 [0.37, 1.33] |
| IFG |  |  |
| *E vs N* |  |  |
| Brain | 0.75 [0.55, 1.04] | 0.62 [0.41, 0.95] |
| Int. | 1.55 [0.99, 2.44] | 1.72 [0.91, 3.25] |
| *F vs P* |  |  |
| Brain | 0.99 [0.72, 1.36] | 1.03 [0.68, 1.57] |
| Int. | 0.95 [0.61, 1.46] | 1.02 [0.55, 1.88] |
| Insula |  |  |
| *E vs N* |  |  |
| Brain | 0.93 [0.63, 1.37] | 0.68 [0.41, 1.14] |
| Int. | 1.19 [0.69, 2.04] | 1.31 [0.60, 2.85] |
| *F vs P* |  |  |
| Brain | 1.08 [0.73, 1.58] | 1.05 [0.63, 1.76] |
| Int. | 0.89 [0.52, 1.52] | 0.89 [0.42, 1.91] |
|  |  |  |
| *E vs N* |  |  |
| Brain | 0.98 [0.69, 1.40] | 1.12 [0.71, 1.78] |
| Int. | 0.95 [0.58, 1.53] | 0.69 [0.35, 1.36] |
| *F vs P* |  |  |
| Brain | 0.97 [0.70, 1.34] | 0.95 [0.62, 1.45] |
| Int. | 1.07 [0.69, 1.66] | 1.08 [0.58, 2.01] |
| mOFC |  |  |
| *E vs N* |  |  |
| Brain | 0.96 [0.82, 1.13] | 0.81 [0.65, 0.99] |
| Int. | 1.18 [0.95, 1.48] | 1.44 [1.05, 1.97] |
| *F vs P* |  |  |
| Brain | 0.98 [0.84, 1.15] | 1.15 [0.93, 1.41] |
| Int. | 1.07 [0.86, 1.33] | 1.08 [0.80, 1.48] |
| STS |  |  |
| *E vs N* |  |  |
| Brain | 0.77 [0.52, 1.14] | 0.56 [0.34, 0.94] |
| Int. | 1.24 [0.72, 2.14] | 1.39 [0.64, 3.02] |
| *F vs P* |  |  |
| Brain | 1.15 [0.80, 1.66] | 0.86 [0.53, 1.38] |
| Int. | 0.98 [0.59, 1.63] | 1.65 [0.81, 3.37] |
| TPJ |  |  |
| *E vs N* |  |  |
| Brain | 0.83 [0.55, 1.25] | 0.73 [0.43, 1.26] |
| Int. | 1.24 [0.70, 2.20] | 0.92 [0.41, 2.07] |
| *F vs P* |  |  |
| Brain | 1.04 [0.70, 1.54] | 0.89 [0.53, 1.50] |
| Int. | 1.07 [0.62, 1.86] | 1.32 [0.61, 2.88] |

Note. Cells correspond to model output for each model in the analysis. E vs N = emotional vs. neutral contrast in the emotional n-back task. F vs P = face vs. place contrast in the emotional n-back task. rACC = rostral anterior cingulate cortex. Amyg. = amygdala. IFG = inferior frontal gyrus. IPS = intraparietal sulcus. mOFC = medial orbitofrontal cortex. STS = superior temporal sulcus. TPJ = temporoparietal junction. Sel = selective profile. Rob = robust profile. Conc = concerning profile.

**Figure S1. Screening procedure.**

**
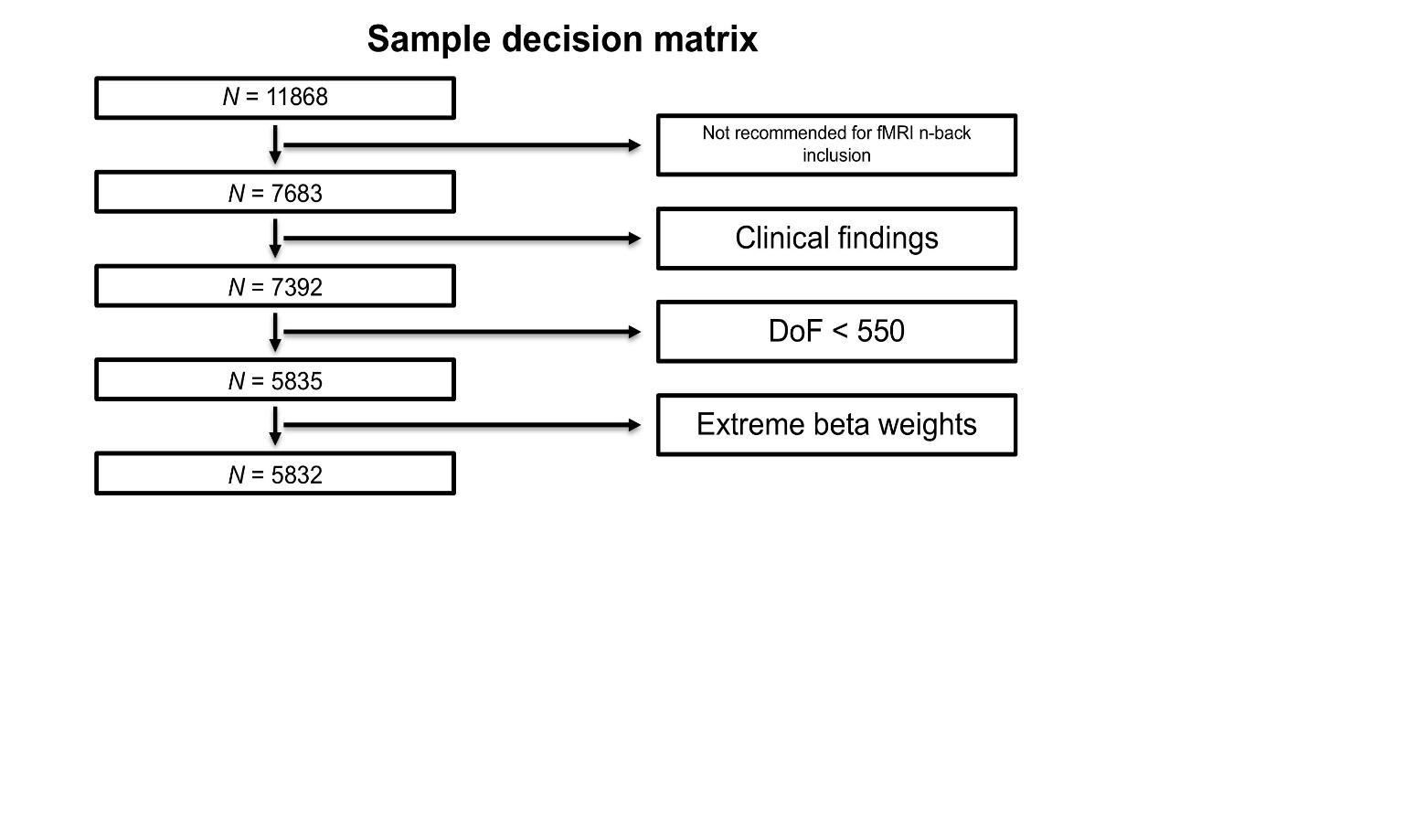
**

Note: Figure overviews the screening criteria applied in the study and the corresponding *N* after applying each criterion. DoF = degrees of freedom.

**Figure S2. Distribution of beta weights after applying exclusion criteria.**

**
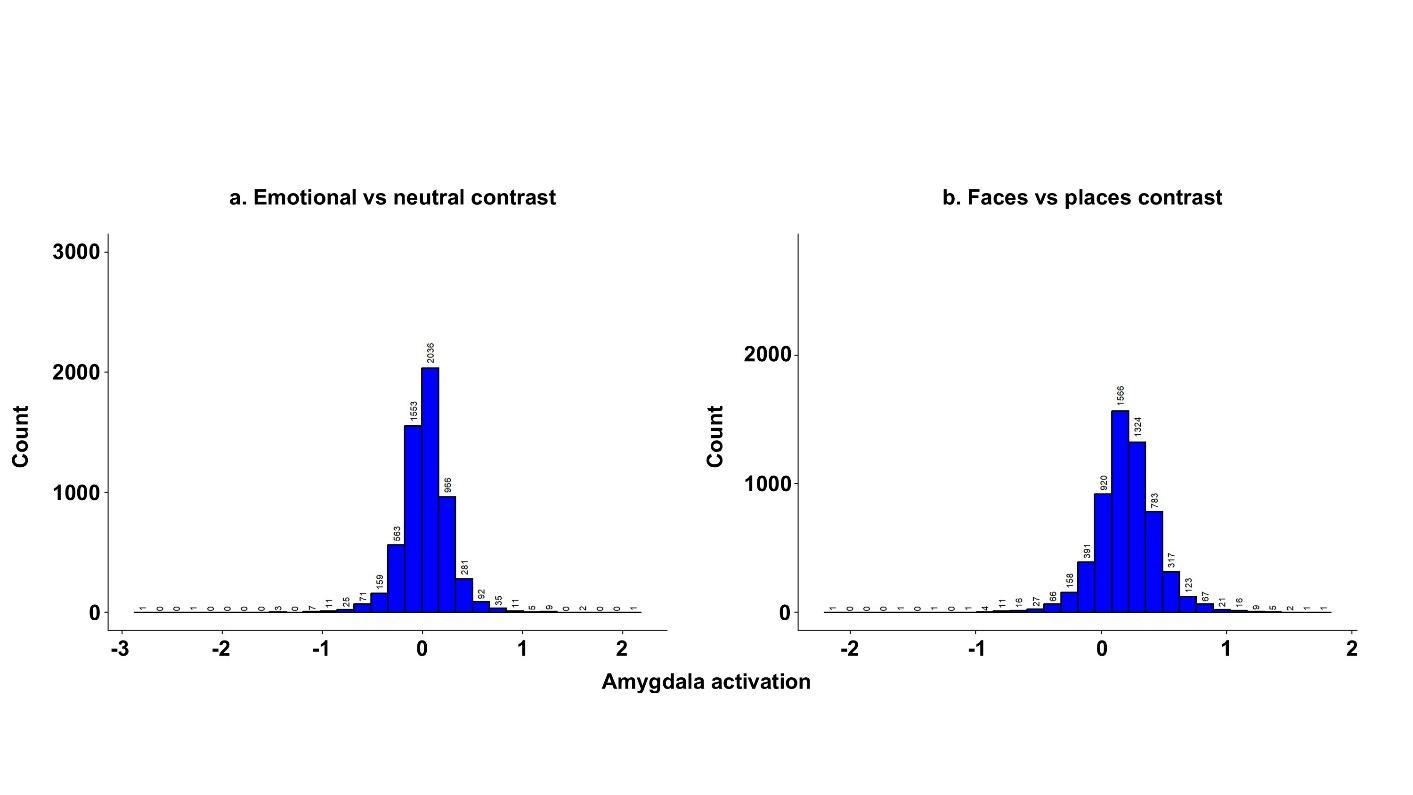
**

Note: Figure displays distributions for amygdala beta weights for the emotional vs neutral (a) and faces vs places (b) contrasts after applying exclusion criteria.

**Figure S3. Distribution of social health outcomes.**

**
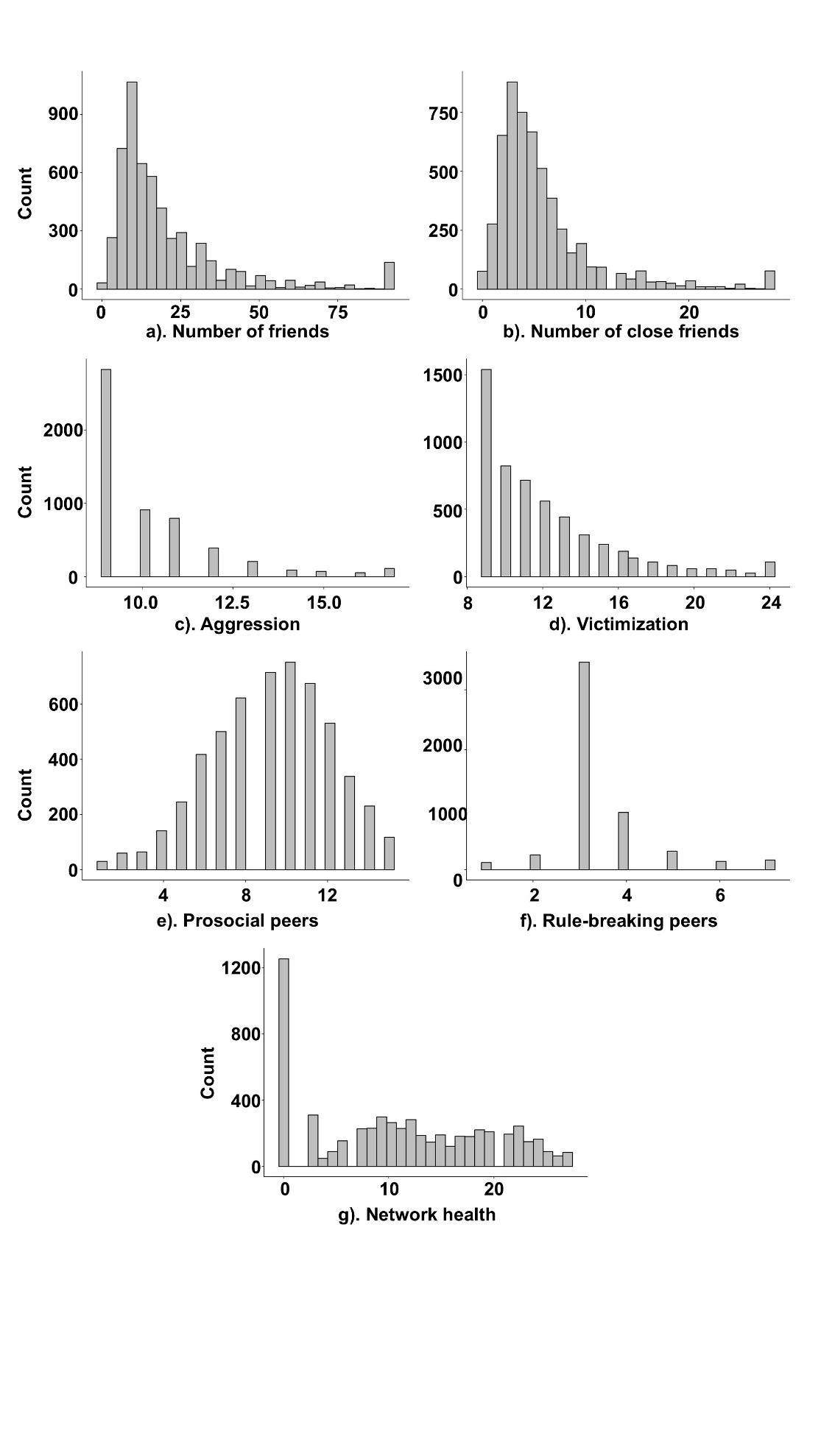
**

Note: Figure displays histograms for the distribution of the number of friends (a), the number of close friends (b), experiences with aggression (c), experiences with victimization (d), the proportion of prosocial peers (e), the proportion of rule-breaking peers (f), and peer network health (g).

**Figure S4. Simulated multinomial regression.**


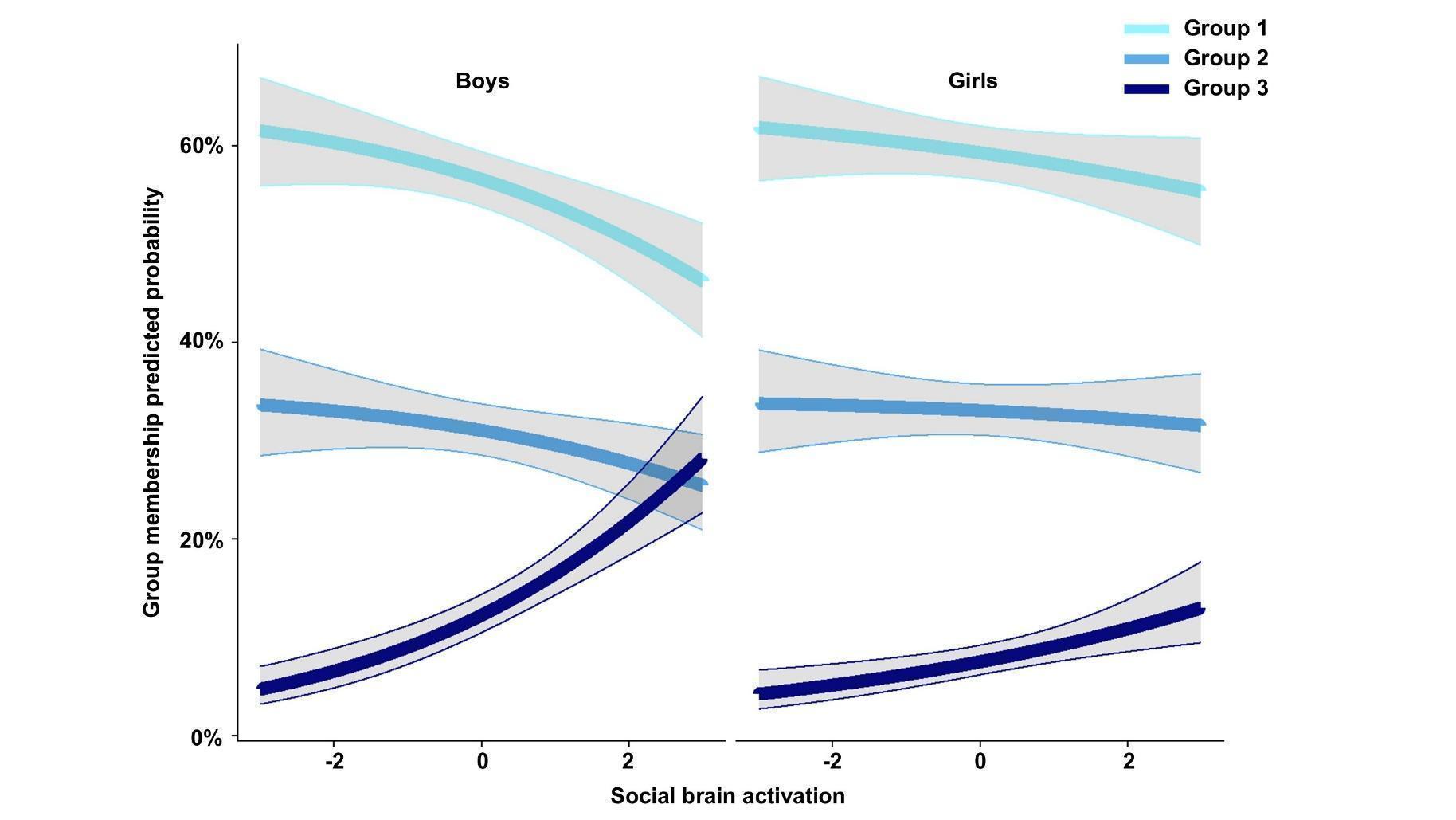


Note: Figure displays the simulated predicted probabilities from a multinomial regression fit using starting values in line with our a-priori hypotheses regarding the associations between sex, social brain activation, and social health profile membership.

**Figure S5. Simulated power curve.**

**
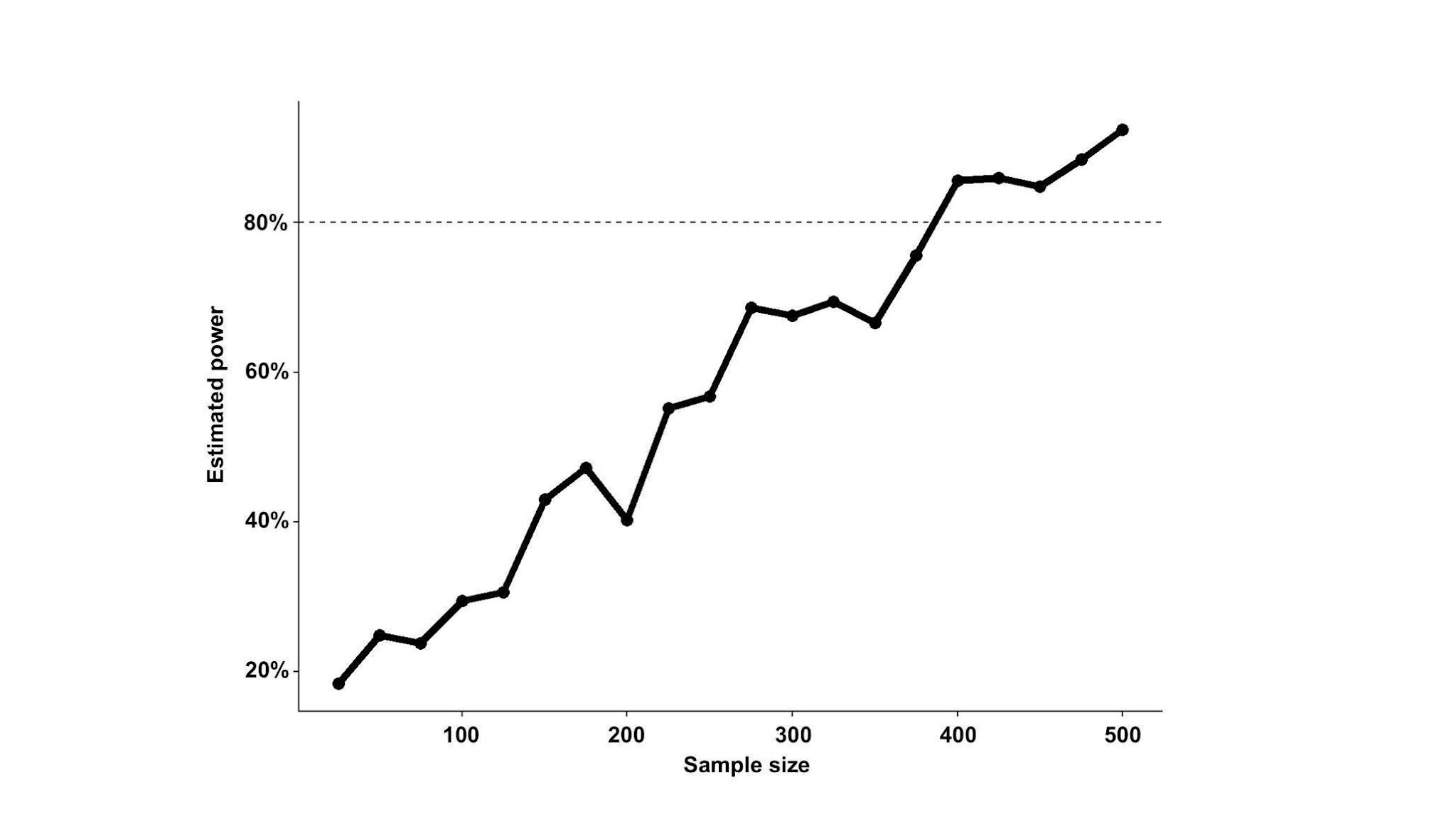
**

Note: Figure displays the simulated power curve for detecting hypothesized effects in samples ranging from *N* = 25 to *N* = 500.

**Figure S6. Co-activation patterns across task contrasts.**

**
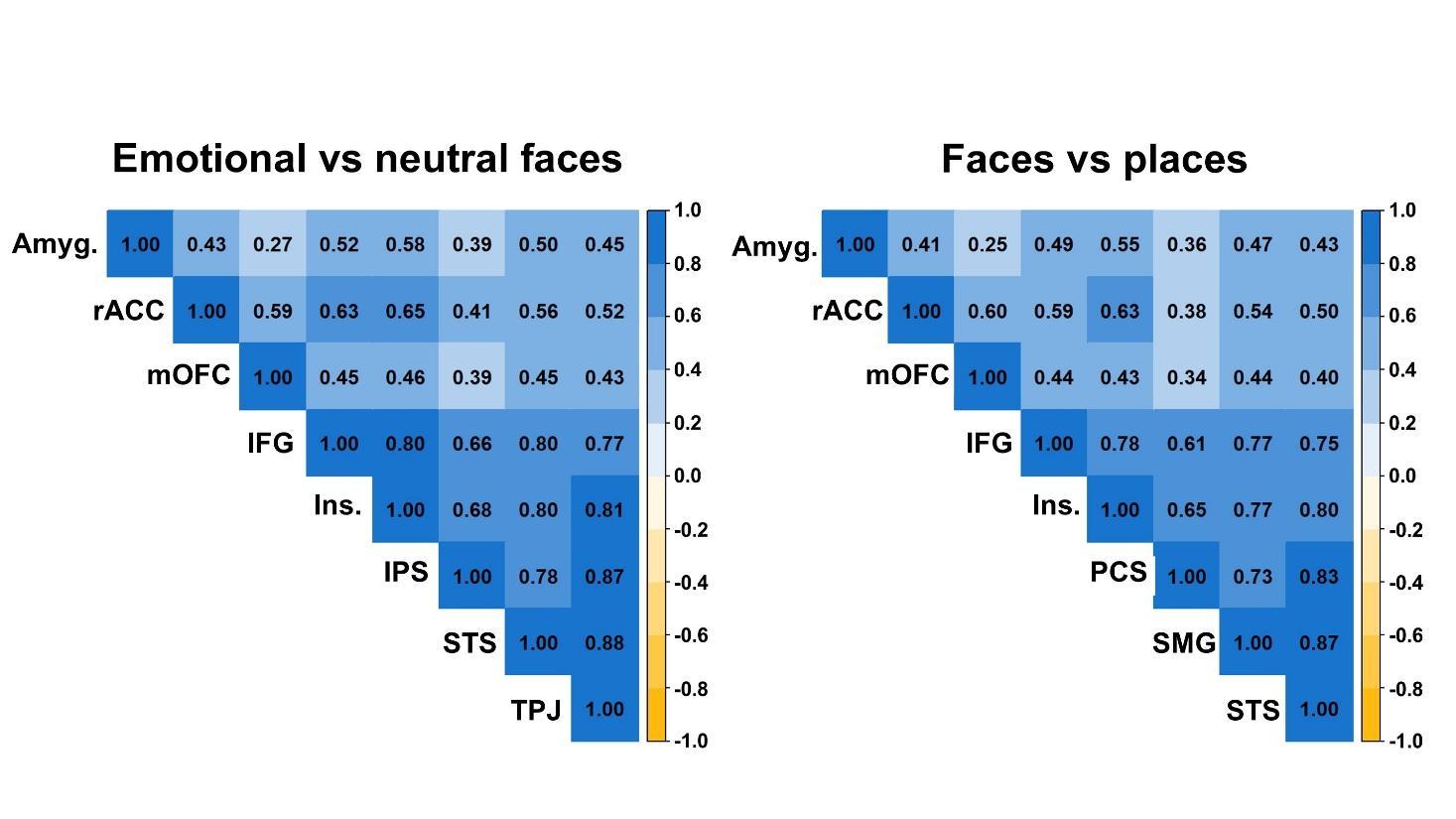
**

Note: Figure displays the bivariate correlations in regional activation among each of the eight social brain regions, separately for the emotional vs neutral (a) and faces vs places (b) contrast in the n-back task. rACC = rostral anterior cingulate cortex. Amyg. = amygdala. IFG = inferior frontal gyrus. IPS = intraparietal sulcus. mOFC = medial orbitofrontal cortex. STS = superior temporal sulcus. TPJ = temporoparietal junction. All correlations were significant at the level of *p* < 0.001.

**Figure S7. Effects of sex on social health profile membership.**

**
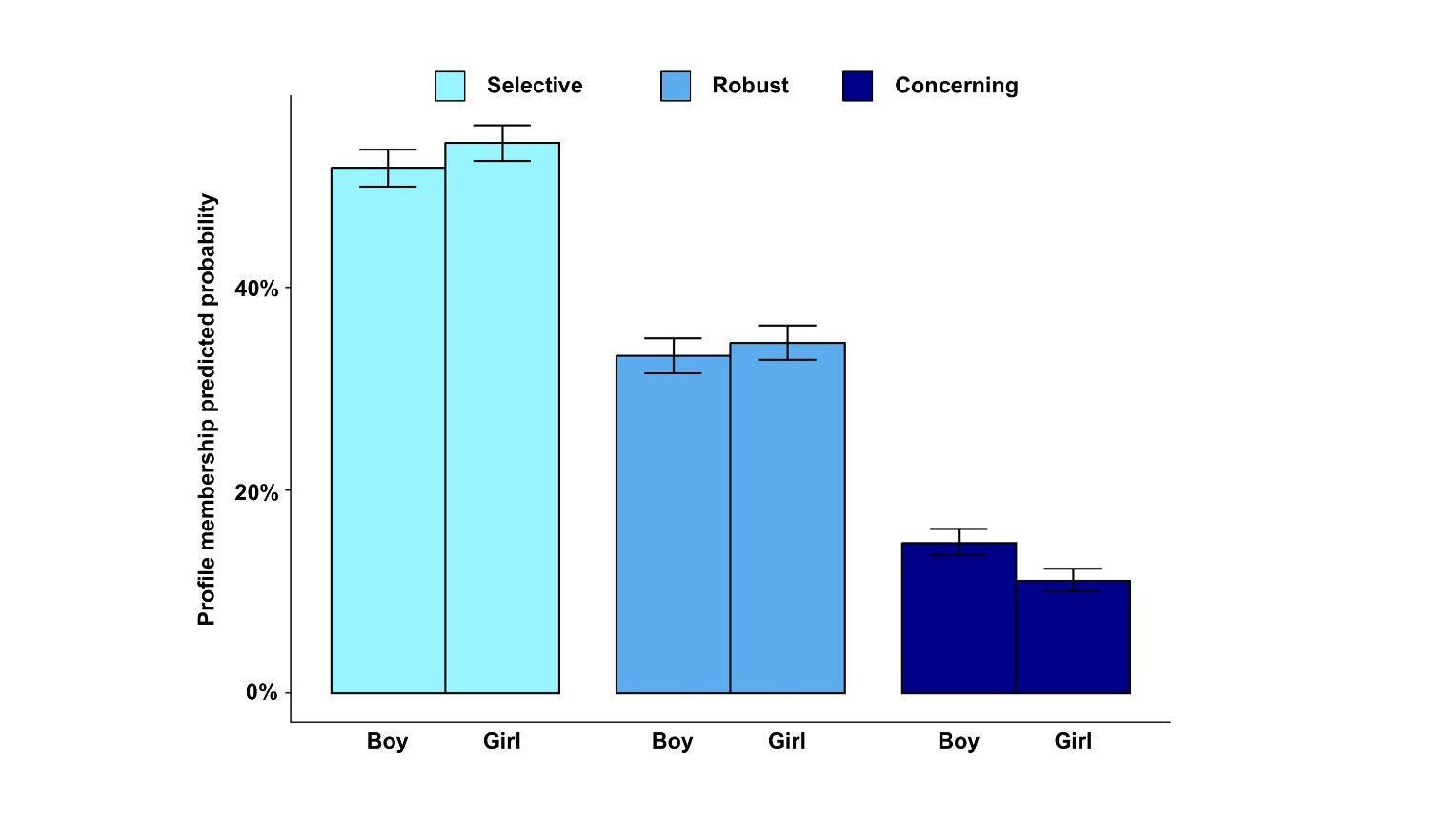
**

Note: Figure presents the predicted probability with confidence interval of membership in each of the three social health profiles as predicted by participant sex.

**Figure S8. Effects from multinomial regressions that did not survive FDR correction.**

**
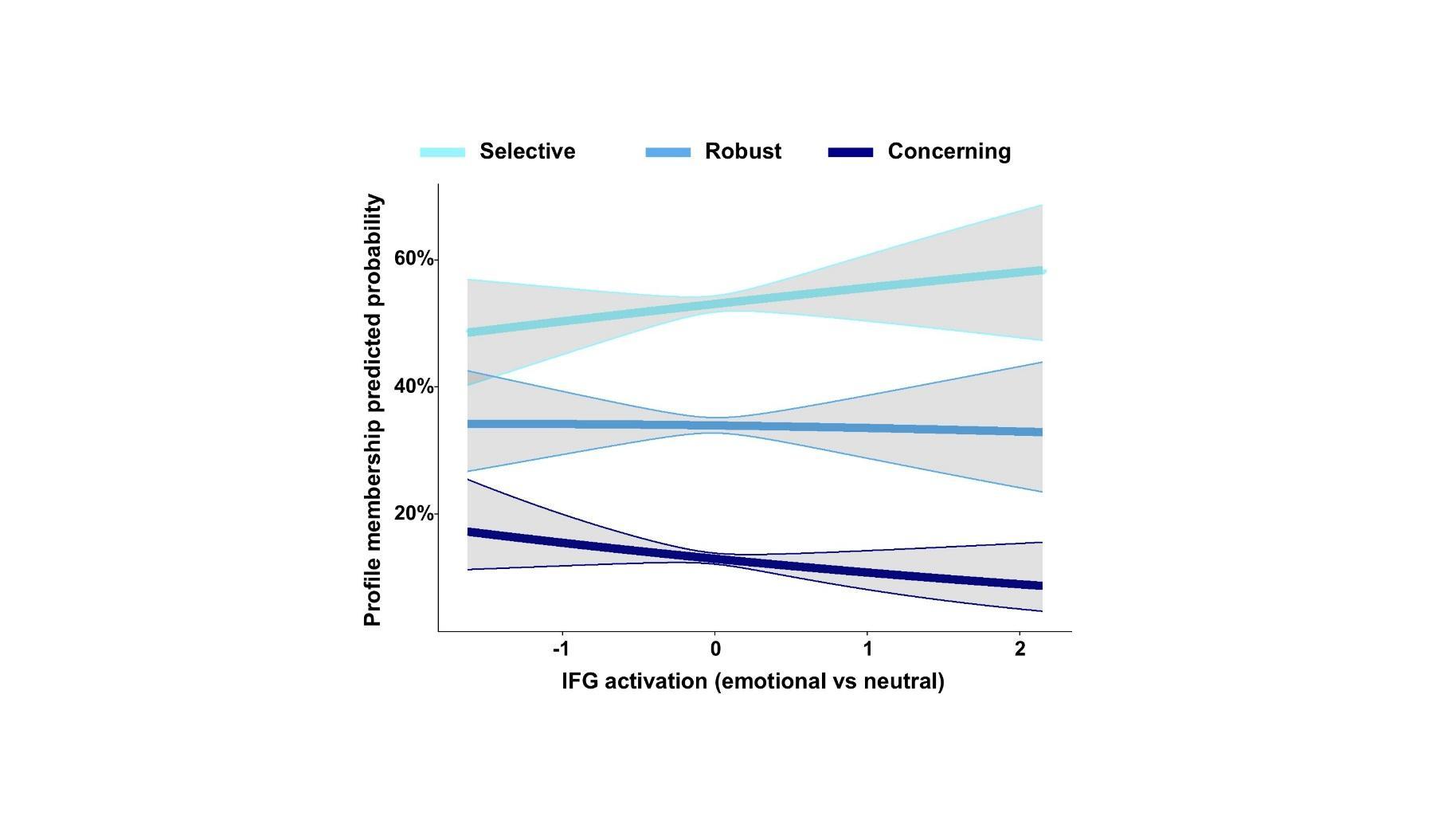
**

Note: Figure shows the main effect of IFG activation in predicting social health profiles. In the multinomial regressions, each index of social brain activity was entered as a predictor of membership in each of the three latent social health profiles alongside sex. Membership in the selective profile was set as the reference. Each line shown in the figure shows the predicted probability (i.e., likelihood on a total range of 0-100%) of a participant belonging to each of the three latent social health profiles across the range of social brain activity.
